# New Chaetoglobosins with Fungicidal Activity from *Chaetomium* sp. UJN-EF006 Endophytic in *Vaccinium bracteatum*

**DOI:** 10.3390/jof11070511

**Published:** 2025-07-07

**Authors:** Luo-Jing Wang, Zong-Yan Ma, Xin-Ling Wang, Kai-Le Wang, Tong Zhang, Rui-Ying Han, Jun-Jiang Li, Jie Bao, Yin-Yin Wang, Hua Zhang

**Affiliations:** 1 School of Biological Science and Technology, University of Jinan, Jinan 250022, China; 2 School of Chemistry and Chemical Engineering, University of Jinan, Jinan 250022, China

**Keywords:** *Chaetomium* sp., chaetoglobosin, phytopathogen, fungicide, *Botrytis cinerea*, *Sclerotinia sclerotiorum*

## Abstract

Nine chaetoglobosins (**1**–**9**) including five previously undescribed ones (**1**–**5**) were obtained from the culture broth of an endophytic fungus (*Chaetomium* sp. UJN-EF006) isolated from the leaves of *Vaccinium bracteatum*. The structures of these fungal metabolites were elucidated by spectroscopic methods including mass spectroscopy, nuclear magnetic resonance, single crystal X-ray crystallography, and electronic circular dichroism. To accelerate the development of novel fungicides, all of the isolated chaetoglobosins were evaluated for their antifungal activity against two crop pathogens, *Botrytis cinerea* and *Sclerotinia sclerotiorum*. The assay results revealed that chaetoglobosins **2**, **6**, **7**, and **9** possessed a significant fungicidal effect against *B. cinerea*, with EC_50_ values all below 10 μg/mL. Particularly, the most potent compound, **7**, was 175- and 96-fold as active as the commercially available fungicides carbendazim (EC_50_ 70.11 μg/mL) and azoxystrobin (EC_50_ 39.02 μg/mL), respectively. A further observation under scanning electron microscope indicated that compound **2** could markedly impair the fungal hyphae of *B. cinerea*. The study demonstrates that the chaetoglobosins had excellent in vitro antifungal activities against *B. cinerea*.

## 1. Introduction

Endophytic fungi (or fungal endophytes) from living plants have received tremendous attention from the scientific community due to their special symbiotic relationship with their hosts [1,2] and are particularly well-known in the literature for their capability to produce diverse bioactive small-molecule natural products [3,4]. These specialized metabolites are believed to possess huge application potential in agriculture to combat crop pests and pathogens [5,6,7] as well as in the pharmaceutical industry to fight against various human diseases [8,9,10]. Therefore, studies on endophytic fungi have emerged as frontline research topics in recent years and are currently on a fast development track.

*Vaccinium bracteatum* Thunb., a native Chinese plant species, is a member of the famous *Vaccinium* genus that has a number of berry trees (e.g., blueberry) with important economic value [11,12]. Our previous investigations into the bioactive constituents of this plant have afforded a large number of compounds including lignans, iridoids, and flavones with various bioactivities [13,14]. *V. bracteatum* in its original habitat is rarely affected by plant pathogens except for *Rhizoctonia solani*, and its leaf extract has been used by the local residents as a food preservative. *Chaetomium globosum* (family Chaetomiaceae, order Sordariales) is a ubiquitous filamentous fungus renowned as both a prolific source of structurally complex secondary metabolites and an effective biocontrol agent against phytopathogens such as mediating antagonism against plant pathogens through secreted chaetoglobosins and underpinning its potential in sustainable agriculture [15]. Initially characterized by its distinctive globose ascomata and coiled peridial hairs, this species has been isolated from diverse ecological niches, including soil, plant material, and crucially as an endophyte in medicinal plants such as *V. bracteatum* (Ericaceae) [16,17], while chaetoglobosins A and B were originally isolated from *Chaetomium globosum* in 1973 by Natori and coworkers [18]. The most important cytochalasan-producing fungi are members of the genera *Chaetomium* and *Aspergillus*, and nearly 200 cytochalasans reported to date (accounting for about 40%) have been isolated from these two genera [19].

Cytochalasans represent a class of structurally diverse fungal metabolites featuring a perhydroisoindolone moiety fused to a macrocyclic ring. There are altogether six groups (about 500 compounds) of cytochalasans in terms of the different amino acids incorporated into the polyketide skeleton. The chaetoglobosins are a group of cytochalasans with a tryptophan unit in the core structure, and 133 chaetoglobosins are included in this section [19]. These chaetoglobosins demonstrate broad-spectrum bioactivities due to their complex molecular architectures, which have attracted significant attention from the chemical and pharmacological scientific communities, and have already been reviewed in recent years from different vantage points such as their chemistry and biology [20], biosynthesis [21], and total synthesis [22]. Plant pathogenic fungi that often cause adverse effects in agricultural production mainly include *Fusarium*, *Alternaria*, *Phytophthora*, etc. *Botrytis cinerea*, a necrotrophic fungal pathogen of global significance, has a wide range of hosts and can cause disease in many economically important crop species such as grapes, strawberries, tomatoes, and ornamental plants [23]. Chemical fungicides currently serve as the dominant approach for plant disease management due to their cost-effectiveness and potent efficacy. Nevertheless, pervasive fungicide overuse has precipitated widespread resistance in phytopathogenic fungi, diminishing the effectiveness of conventional agents. This necessitates the development of novel, high-performance fungicides to resist resistance and mitigate agricultural disease burdens. In this study, we conducted an investigation into the inhibitory effects of chaetoglobosins on *Botrytis cinerea*, aiming to offer a reliable and safe natural agent for effectively managing postharvest decay in tomatoes.

## 2. Materials and Methods

### 2.1. General Experimental Procedures

Specific optical rotation data were measured on a Rudolph VI polarimeter (Rudolph Research Analytical, Hackettstown, NJ, USA). UV and ECD (electronic circular dichroism) spectra were acquired on a Chirascan circular dichroism spectrometer (Applied Photophysics, Surrey, UK). NMR experiments were performed on a Bruker Avance DRX600 spectrometer (Bruker BioSpin AG, Fallanden, Switzerland) and referenced to residual solvent peaks (CD_3_OD: *δ*_H_ 3.31, *δ*_C_ 49.00; CDCl_3_: *δ*_H_ 7.26, *δ*_C_ 77.16). HR-ESIMS spectra were recorded on an Agilent 6545 Q-TOF mass spectrometer (Agilent Technologies Inc., Waldbronn, Germany). ESIMS analyses were performed on an Agilent 1260-6460 Triple Quad LC-MS instrument (Agilent Technologies Inc., Waldbronn, Germany). HPLC analyses and separations were conducted on an Agilent 1260 series LC instrument (Agilent Technologies Inc., Waldbronn, Germany) coupled to a SilGreen-C_18_ column (10 mm × 250 mm, Greenherbs Science and Technology, Beijing, China). Conventional column chromatography (CC) was performed on D101-macroporous absorption resin (Sinopharm Chemical Reagent Co. Ltd., Shanghai, China), reversed-phase C_18_ silica gel (Merck KGaA, Darmstadt, Germany), Sephadex LH-20 (GE Healthcare Bio-Sciences AB, Uppsala, Sweden), and silica gel (300–400 mesh; Qingdao Marine Chemical Co. Ltd., Qingdao, China). All solvents used for CC were of analytical grade (Tianjin Fuyu Fine Chemical Co. Ltd., Tianjin, China), and solvents used for HPLC were of HPLC grade (Oceanpak Alexative Chemical Ltd., Goteborg, Sweden). Pre-coated silica gel GF_254_ plates (Qingdao Marine Chemical Co. Ltd., Qingdao, China) were used for TLC monitoring. All mixed solvent systems used in the experiments were in the form of *v*/*v* unless otherwise specified. A Tecan Spark10M microplate reader (Salzburg, Austria) and scanning electron microscope (Zeiss, Gemini300, Jena, Germany) were used in the biological tests.

### 2.2. Fungal Source and Culture Conditions

The fungal species was isolated from the fresh leaves of *Vaccinium bracteatum*. Fresh plant leaves were collected and surface-sprayed with 75% ethanol for sterilization, which were cut using a sterilized scalpel. Then, the incised leaves were placed onto pre-prepared PDA plates. The plates were incubated at 28 °C, and the colonies were monitored and transferred to fresh PDA plates for fungal isolates. The ITS sequencing information and phylogenetic tree analysis of UJN-EF006, shown in the Supplementary Materials (Figure S45), indicated a 97.00% similarity to *Chaetomium subglobosum*. The fermentation and extraction of *Chaetomium* sp. UJN-EF006 was the same as that previously described [17].

The plant pathogens *Botrytis cinerea* and *Sclerotinia sclerotiorum* (Lib.) de Bary were acquired from Shandong Academy of Pesticide Science, and the pure cultures with 20% (*v*/*v*) glycerol were stored at −20 °C as spores and mycelial fragments. The two fungal pathogens were cultured on potato dextrose agar (PDA: potato extract 20 g/L, dextrose 20 g/L, agar 15 g/L, and pH 7.2) at 25 °C in the dark.

### 2.3. Extraction and Isolation

The EtOAc partition (700 g) generated from the EtOH extract of the fungal materials was acquired as recorded in a recent report [17] and then fractionated by D101 macroporous resin CC (EtOH-H_2_O, 30%, 50%, 80% and 90%) to obtain four fractions. The 50% EtOH eluate (150 g) was subsequently separated on a silica gel column eluted with step-gradient CH_2_Cl_2_–MeOH (100:0 to 50:1) to produce eight subfractions (Fr.1–Fr.8). Fr.2 was chromatographed on RP-C_18_ silica gel CC (MeOH-HO, 65% to 90%) to produce five subfractions, and the fifth one was further purified by semi-preparative HPLC (65% MeCN-H_2_O) to afford **4** (1.6 mg, *t*_R_ = 14.0 min). Both Fr.4 and Fr.7 were separated by Sephadex LH-20 CC (in CH_2_Cl_2_–MeOH) to afford three respective subfractions, Fr.4-1 to Fr.4-3 and Fr.7-1 to Fr.7-3. Fr.4-1 was separated by silica gel CC (CH_2_Cl_2_-acetone, 50:1 to 10:1) to yield three subfractions, and the second one was further purified by semi-preparative HPLC (55% MeCN-H_2_O) to afford **2** (45.0 mg, *t*_R_ = 12.3 min), while the third one was purified by semi-preparative HPLC (45% MeCN-H_2_O) to afford **9** (15.0 mg, *t*_R_ = 27.0 min). Fr.7-2 was fractionated by silica gel CC (CH_2_Cl_2_–MeOH, 200:1 to 20:1) to afford four subfractions, and the second one was further purified by semi-preparative HPLC (45% MeCN-H_2_O) to afford **1** (12.6 mg, *t*_R_ = 17.0 min). Fr.5 was separated by silica gel CC (CH_2_Cl_2_–MeOH, 200:1 to 30:1) to yield six subfractions (Fr.5-1 to Fr.5-6), and Fr.5-4 was separated by Sephadex LH-20 CC (in CH_2_Cl_2_–MeOH) to afford two further subfractions (Fr.5-4-1 and Fr.5-4-2). Fr.5-4-1 was purified by semi-preparative HPLC (52% MeCN-H_2_O) to afford **7** (12.5 mg, *t*_R_ = 21.5 min). Fr.6 was separated by silica gel CC (CH_2_Cl_2_–MeOH, 400:1 to 15:1) to yield four subfractions (Fr.6-1 to Fr.6-4), and Fr.6-3 and Fr.6-4 were separated by Sephadex LH-20 CC (in CH_2_Cl_2_–MeOH) to afford four respective subfractions Fr.6-3-1 to Fr.6-3-4 and Fr.6-4-1 to Fr.6-4-4. Fr.6-3-2 was chromatographed on a RP-C_18_ silica gel column (MeOH-H_2_O, 40% to 60%) to produce three subfractions (Fr.6-3-2-1 to Fr.6-3-2-3), and Fr.6-3-2-2 was further purified by semi-preparative HPLC (45% MeCN-H_2_O) to afford **5** (1.5 mg, *t*_R_ = 28.5 min). Fr.6-4-2 was separated by silica gel CC (CH_2_Cl_2_–acetone, 15:1 to 10:1) and semi-preparative HPLC (50% MeCN-H_2_O) to give **3** (2.1 mg, *t*_R_ = 40.0 min) and **6** (27.4 mg, *t*_R_ = 44.0 min). Fr.6-4-4 was chromatographed on a RP-C_18_ silica gel column (MeOH-H_2_O, 35% to 50%) to produce three subfractions (Fr.6-4-4-1 to Fr.6-4-4-3), and Fr.6-4-4-2 was further purified by semi-preparative HPLC (50% MeCN-H_2_O) to afford **8** (12.2 mg, *t*_R_ = 29.0 min).The isolation procedures are displayed in flowchart in the Supplementary Materials (Figure S44).

19-Oxo-deoxaphomin B (Compound **1**): white solid; [*α*]_D_^25^ +80 (*c* 1.0, MeOH); UV (MeOH) *λ*_max_ (log *ε*) 225 (4.40) nm; ECD (0.13 mg/mL, MeOH) *λ*_max_ (Δ*ε*) 240 (−2.95), 232 (−0.64), 200 (+55.34) nm; ^1^H and ^13^C NMR data (in CD_3_OD) see Table 1; (+)-ESIMS *m*/*z* 508.3 [M + H]^+^; (+)-HR-ESIMS *m*/*z* 508.2694 [M + H]^+^ (C_30_H_38_NO_6_, calcd. 508.2694).

19-*O*-acetyl-dehydrochaetoglobosin F (Compound **2**): light yellow solid; [*α*]_D_^25^ +36 (*c* 0.50, MeOH); UV (MeOH) *λ*_max_ (log *ε*) 220 (4.96) nm; ECD (0.13 mg/mL, MeOH) *λ*_max_ (Δ*ε*) 325 (+4.80), 260 (−53.35), 220 (+59.38), 205 (+25.0) nm; ^1^H and ^13^C NMR data (in CDCl_3_) see Table 1; (−)-ESIMS *m*/*z* 605.3 [M + Cl]^−^; (+)-HR-ESIMS *m*/*z* 571.2798 [M + H]^+^ (C_34_H_39_N_2_O_6_, calcd. 571.2803).

19-*O*-acetyl-21,22-dihydro-22-methoxychaetoglobosin A (Compound **3**): light yellow solid; [*α*]_D_^25^ −40 (*c* 0.10, MeOH); UV (MeOH) *λ*_max_ (log *ε*) 222 (4.16) nm; ECD (0.13 mg/mL, MeOH) *λ*_max_ (Δ*ε*) 295 (−4.69), 205 (+6.33) nm; ^1^H and ^13^C NMR data (in CDCl_3_) see Table 1; (+)-ESIMS *m*/*z* 603.1 [M + H]^+^; (+)-HR-ESIMS *m*/*z* 603.3060 [M + H]^+^ (C_35_H_43_N_2_O_7_, calcd. 603.3065).

19-*O*-acetyl-21,22-dihydro-22-ethoxychaetoglobosin A (Compound **4**): yellow solid: [*α*]_D_^25^ −93 (*c* 0.10, MeOH); UV (MeOH) *λ*_max_ (log *ε*) 222 (4.59) nm; ECD (0.15 mg/mL, MeOH) *λ*_max_ (Δ*ε*) 295 (−8.91), 210 (−0.20) nm; ^1^H and ^13^C NMR data (in CDCl_3_) see Table 1; (+)-ESIMS *m*/*z* 617.3; (+)-HR-ESIMS *m*/*z* 617.3222 [M + H]^+^ (C_36_H_44_N_2_O_7_, calcd. 617.3221).

19-*O*-acetyl-21,22-dihydro-21-methoxychaetoglobosin A (Compound **5**): light yellow solid; [*α*]_D_^25^ −140 (*c* 0.75, MeOH); UV (MeOH) *λ*_max_ (log *ε*) 220 (4.82) nm; ECD (0.19 mg/mL, MeOH) *λ*_max_ (Δ*ε*) 305 (−15.63), 215 (+22.0) nm; ^1^H and ^13^C NMR data (in CDCl_3_) see Table 1; (−)-ESIMS *m*/*z* 637.2 [M + Cl]^−^; (+)-HR-ESIMS *m*/*z* 620.3334 [M + NH_4_]^+^ (C_35_H_46_N_3_O_7_, calcd. 620.3330).

### 2.4. X-Ray Crystallographic Analysis

The crystallographic data for compound **1** have been deposited at the Cambridge Crystallographic Data Center (CCDC) as a supplementary publication (registration no. CCDC 2339221), and copies of the data can be obtained free of charge from the CCDC (12 Union Road, Cambridge CB2 1EZ, UK (Fax: Int. + 44(0) (1223) 336 033; email: deposit@ccdc.cam.ac.uk).

Crystallographic data for **1**: C_30_H_36_NO_6_, M = 506.60, *a* = 78930 (2) Å, *b* = 13.8373 (3) Å, *c* = 12.5689 (2) Å, *β* = 97.9440 (10) Å, *V* = 1359.57 (5) Å^3^, *T* = 150 K, space group *P*2_1_, *Z* = 2, *μ* (Cu Kα) = 0.694 mm^−1^, 8807 reflections measured, 4650 independent reflections (*R_int_* = 0.0268). The final *R*_1_ values were 0.0668 (*I* > 2*σ* (*I*)). The final *R*_1_ values were 0.0671 (all data). The final *wR* (*F*^2^) values were 0.1525 (*I* > 2*σ* (*I*)). The final *wR* (*F*^2^) values were 0.1531 (all data). The goodness of fit on *F*^2^ was 1.097. Flack parameter = 0.08 (7) [24].

### 2.5. ECD Calculation

The absolute configuration of compound **2** was first subjected to random conformational analysis with the MMFF force field, and determined by time-dependent density functional theory (TD-DFT) based electronic circular dichroism calculation with Gaussian 09 software at the CAM-B3LYP/6-311G (d, p) level in vacuo. The ECD spectrum of each conformer was simulated by SpecDis 1.71 with a half-bandwidth of 0.3 eV, and the final ECD spectra of the target molecules were obtained according to the Boltzmann calculated contribution of each conformer with UV wavelength correction [13].

### 2.6. In Vitro Antifungal Evaluation

The preliminary anti-phytopathogenic effects of all of the isolated chaetoglobosins against *B. cinerea* and *S. sclerotiorum* at the concentration of 10 μg/mL were screened according to a mycelial growth inhibition method [25]. Briefly, each tested compound was dissolved in DMSO and then thoroughly mixed at about 55 °C in PDA medium sterilized to obtain a series of concentrations (0, 0.5, 2.5, 5.0 μg/mL), while azoxystrobin and carbendazim were used as a positive control. After solidification of the PDA medium, the mycelial dishes (5.00 mm) of phytopathogenic fungi were inoculated on the center of the PDA plates and incubated at 25 °C in the dark. Three replicates were performed for each parallel experiment. The diameters (mm) of the inhibition zones were measured by the cross-bracketing method after three days. The growth inhibition rates were calculated when the mycelia in the blank control group grew to the edge of the Petri dish according to the following formula: mycelial growth inhibition (%) = [(d_c_ − d_t_)/(d_c_ − 5 mm)] × 100%, where d_c_ and d_t_ are the average diameters of the fungal colony in the black control and treatment groups, respectively. Standard deviation (SD) values were calculated based on the inhibition data obtained from triplicate experiments for each assay.

Chaetoglobosins with good inhibitory activity were further evaluated to determine their median effective concentration (EC_50_) values using established procedures [26]. The values were calculated from the toxicity regression equation (*y* = a*x* + b) using Microsoft Excel software by the least-squares method, with the logarithmic values of concentrations as the independent variable (x) and the probit values of the inhibition rates as the dependent variable (y).

### 2.7. Scanning Electron Microscopy (SEM)

The effect of the new compound **2** on the morphology of *B. cinerea* was observed via SEM. The fresh mycelia of *B. cinerea* were inoculated into the potato dextrose broth medium and cultured at 25 °C for 12 h. The stock solution of compound **2** was added to the medium to achieve concentrations of 2.5 and 5 μg/mL and then cultured at 25 °C for 48 h. All of the samples were fixed with 2.5% glutaraldehyde stationary liquid at 4 °C for 12 h and then washed thrice with 0.1 M phosphate buffer (pH 7.2) for 5 min each time. After that, each sample was dehydrated in an ethanol gradient of 30%, 50%, 70%, 90%, 100% for 15 min each, and finally washed with 100% *t*-butanol twice [27]. Finally, the sample was freeze-dried, coated with gold at 2.0 kv, and visualized under a scanning electron microscope (Zeiss, Gemini300, Germany).

## 3. Results and Discussion

### 3.1. Structure Characterization of the Isolated Compounds

**Figure 1:**
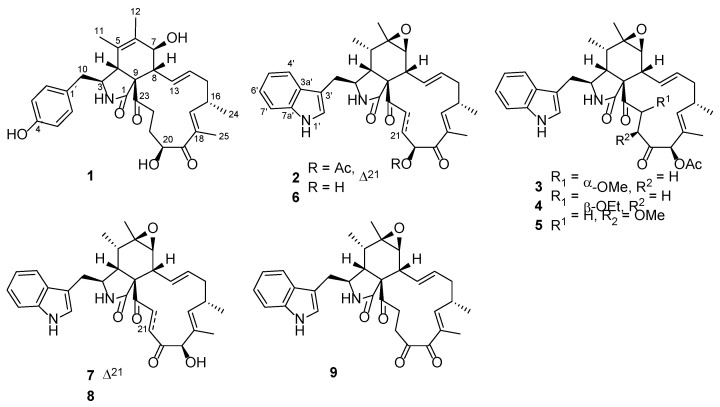
Chemical structures of compounds **1**–**9**.

An endophytic fungal strain was isolated from the surface-sterilized fresh leaves of the host plant *V. bracteatum* (Ericaceae). Based on morphological and molecular characterization (ITS sequencing information is presented in the supporting material), this strain was identified as *Chaetomium globosum*. Due to its rich metabolite profile, according to HPLC analysis and a subsequent intensive fractionation on the EtOH extract of this fungus, this resulted in the separation and structural characterization of an array of chaetoglobosins (**1**–**9**) including five new ones (**1**–**5**) (Figure 1). Of particular note, compounds **2**, **6**, **7**, and **9** showed significant inhibitory activity against a common crop pathogen Botrytis cinerea, with a much better effect than the positive controls azoxystrobin and carbendazim. Details of the isolation, structure characterization, and anti-phytopathogenic evaluation of these interesting fungal metabolites are presented in the current paper.

**Figure 2:**
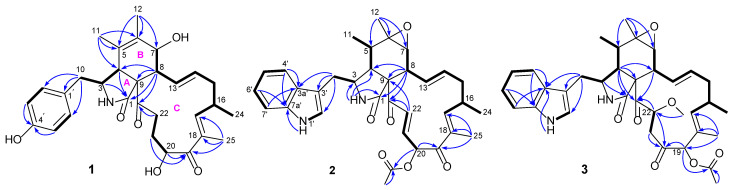
Key ^1^H-^1^H COSY (bold lines) and HMBC (arrows) correlations of compounds **1**–**3**.

Compound **1** was isolated as a white solid and showed a protonated molecular ion in the HR-ESIMS analysis at *m*/*z* 508.2694 (calcd. 508.2694), corresponding to the molecular formula C_30_H_37_NO_6_. Inspection of the NMR data (in CD_3_OD, Table 1) for **1** revealed the presence of three carbonyls (*δ*_C_ 177.1, 205.5, 211.2), a 4-hydroxyphenyl unit (*δ*_C_ 129.1, 131.4 (2C), 116.4 (2C), 157.4; *δ*_H_ 6.74, 6.93 (both d, *J* = 8.6 Hz, 2H)), a tetra-substituted double bond (*δ*_C_ 127.7, 134.4), a tri-substituted double bond (*δ*_C_ 150.5, 137.0; *δ*_H_ 6.31 (m)), a di-substituted *trans* double bond (*δ*_C_ 129.4, 136.1; *δ*_H_ 6.28 (ddd, *J* = 15.3, 10.0, 2.0 Hz), 5.23 (ddd, *J* = 15.3, 11.0, 2.9 Hz)), a *sp*^3^ quaternary carbon (*δ*_C_ 63.8), six *sp*^3^ methines [*δ*_C_ 34.6, 50.0, 52.6, 60.5, 70.0, 71.9; *δ*_H_ 2.13 (dd, *J* = 10.0, 9.7 Hz), 2.82 (m), 2.94 (brs), 3.33 (dd, *J* = 9.8, 5.5 Hz), 3.81 (brd, *J* = 9.7 Hz), 4.91 (dd, *J* = 7.8, 4.3 Hz)], four *sp*^3^ methylenes [*δ*_C_ 32.0, 38.2, 42.0, 42.2; *δ*_H_ 1.85 (m), 1.93 (m), 2.08 (ddd, *J* = 13.6, 12.1, 11.0 Hz), 2.18 (dd, *J* = 13.4, 9.8 Hz), 2.48 (m), 2.66 (dd, *J* = 13.4, 5.5 Hz), 2.91 (ddd, *J* = 19.6, 9.1, 5.4 Hz), 3.03 (dt, *J* = 19.6, 5.7 Hz)], and four methyls [*δ*_C_ 12.4, 14.8, 17.4, 20.2; *δ*_H_ 1.07 (d, *J* = 6.7 Hz), 1.28 (brs), 1.64 (d, *J* = 1.6 Hz), 1.83 (d, *J* = 1.3 Hz)]. These NMR spectroscopic features resembled those for the chaetoglobosins, which are a well-known class of fungal metabolites in the literature [28]. Further examination of the 2D NMR data (Figure 2) confirmed the aforementioned deduction, revealing key ^1^H-^1^H COSY correlations of H_2_-10/H-3/H-4, H-7/H-8/H-13/H-14/H_2_-15/H-16(H_3_-24)/H-17, and H-20/H_2_-21/H_2_-22 as well as diagnostic HMBC signals from H-3 and H-4 to C-1, H_3_-11 to C-4, C-5, and C-6, H_3_-12 to C-5, C-6, and C-7, H-8 to C-1, C-4, C-9, and C-23, H_3_-25 to C-17, C-18, and C-19, H-20 and H-21 to C-19, and H_2_-22 to C-23, which corroborated the chaetoglobosin core of **1** as shown (Figure 2). In addition, the connection between the 4-hydroxyphenyl group and the tricyclic chaetoglobosin core was confirmed by the HMBC correlations from H_2_-10 to C-1′ and C-2′(6′). Finally, the remaining unassigned elements and the chemical shits for C-7 (*δ*_C_ 70.0) and C-20 (*δ*_C_ 71.9) supported the locations of 7-OH and 20-OH. The planar structure of **1** was thus unambiguously established, and it is interesting to note that most formerly reported chaetoglobosins incorporate an indole fragment while compound **1** bears a benzene unit.

**Figure 3:**
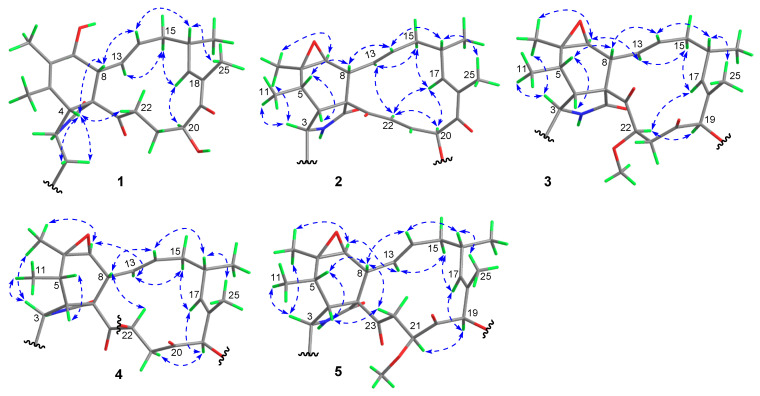
Key NOESY correlations (double-headed dashed arrows) of compounds **1**–**5**.

**Figure 4:**
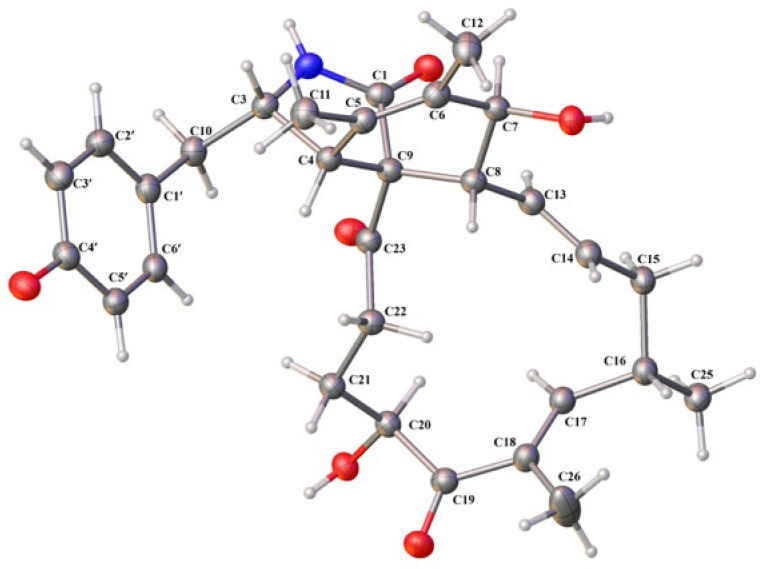
X-ray crystallographic structure of compound **1**.

The relative configuration of **1** was assigned on the basis of analyzing NOESY data (Figure 3) and proton couplings. The correlation of H-4/H-8 indicated that they were in a *quasi* 1,3-diaxial relationship and thus coplanar in ring B, and the correlations of H-4 with H_2_-10 and H-22 (*δ*_H_ 3.03) suggested that the CH_2_-10 and –COCH_2_– (C-23/C-22) groups were co-facial with H-4 in ring A. Then, H-7 was assigned to be on the opposite side of H-8 in ring B according to the large *J*_7,8_ value (9.7 Hz, *quasi* 1,2-diaxial relationship). Afterward, the correlation cascades of H-8/H-14/H-16/H_3_-25 and H-13/H-15 (*δ*_H_ 2.08)/H-18/H-20 established the geometries of double bonds (Δ^13^ and Δ^17^) and configurations of chiral centers (C-16 and C-20), as shown in Figure 3. To corroborate the structure of **1**, we tried several solvent systems to culture suitable single crystals for X-ray crystallographic analysis, and this effort was eventually rewarded by the successful acquisition of a high-quality crystal in MeOH–H_2_O (10:1). The X-ray crystallographic structure of **1** is shown in Figure 4, and its absolute stereochemistry was also unequivocally assigned as 3*S*,4*R*,7*S*,8*R*,9*R*,13*E*,16*S*,17*E*,20*S* (flack parameter = 0.08 (7)).

Compound **2** was assigned the molecular formula C_34_H_38_N_2_O_6_ based on its HR-ESIMS ion at *m*/*z* 571.2798 ([M + H]^+^, calcd. 571.2803). Analysis of the NMR data (in CDCl_3_, Table 1) for **2** revealed a high similarity to those for its cometabolite chaetoglobosin F (**6**) [29], and the major difference was attributed to the appearance of signals for an acetoxy group (*δ*_C_ 169.9, 19.8; *δ*_H_ 2.21 (s)) and a *E*-double bond (*δ*_C_ 132.0, 136.8; *δ*_H_ 6.50 (dd, *J* = 15.3, 10.7 Hz), 7.44 (d, *J* = 15.3 Hz)) in **2** instead of those for two methylenes in **6**. Further interpretation of the 2D NMR ^1^H-^1^H COSY and HMBC data (Figure 2) confirmed the classical chaetoglobosin framework for **2** as drawn, with key COSY correlations of H_2_-10/H-3, H-4/H-5/H_3_-11, H-7/H-8/H-13/H-14/H_2_-15/H-16(H_3_-24)/H-17, H-20/H-21/H-22, H-1′/H-2′, and H-4′/H-5′/H-6′/H-7′ as well as pivotal HMBC signals from H-2 to C-1, C-3 and C-4, H_3_-12 to C-5, C-6, and C-7, H-8 to C-1, C-4, C-9, and C-23, and H_3_-25 to C-17, C-18, and C-19. Particularly, the HMBC correlations from H-22 to C-9 and C-23 corroborated the presence of Δ^21^, and those from H-20 to C-19 and the acetoxy carbonyl carbon, together with the markedly downfield shifted H-20 signal (6.38 ppm, compared with that of **6**), strongly supported the structural evolution from **6** to **2**. The relative configuration of **2** was finally established to be identical with that of **6** based on a careful NMR comparison (especially analysis of proton couplings) and inspection of the NOESY data (Figure 3). The critical NOESY cross-peaks included H-3/H_3_-11, H-3/H_3_-12, and H_3_-12/H-7, and the correlation cascades comprised H-4/H-5/H-8/H-14/H-16/H_3_-25 and H-13/H-15 (*δ*_H_ 2.19)/H-17/H-20/H-22. Moreover, the NOESY signals of H-22 with H-13 and H-17 were also observed. Compound **2** was thus characterized as 19-*O*-acetyl-dehydrochaetoglobosin F.

Compound **3** was assigned the molecular formula C_35_H_42_N_2_O_7_ by HR-ESIMS analysis at *m*/*z* 603.3060 ([M + H]^+^, calcd. 603.3065), with 32 mass units (CH_4_O) more than 19-*O*-acetylchaetoglobosin A [29], indicative of a methanol adduct of the latter. Inspection of the NMR data (in CDCl_3_, Table 1) for **3** confirmed this hypothesis, revealing extra resonances (*δ*_C_ 42.7, 59.0, 80.8; *δ*_H_ 2.65 (dd, *J* = 15.5, 10.1 Hz), 2.94 (dd, *J* = 15.5, 1.7 Hz), *δ*_H_ 3.48 (s), 4.77 (dd, *J* = 10.1, 1.7 Hz)) for a –CH_2_CH(OMe)– fragment rather than those for the Δ^21^ double bond in 19-*O*-acetylchaetoglobosin A, which was further evidenced by the diagnostic ^1^H-^1^H COSY correlations of H_2_-21/H-22 and the HMBC correlation from the methoxy protons to C-22 (Figure 2). The location of this fragment was supported by the HMBC correlations from H-22 to C-23 and from H_2_-21 to C-20. The planar structure of **3** was further secured by careful examination of the full 2D NMR data (Figure 2). Finally, the relative configuration of **3** was established by analysis of the NOESY data (Figure 3). To be specific, most NOESY cross-peaks of **3** were consistent with those of **2**, indicating common stereochemistries at most chiral centers (C-3, C-5, C-6, C-7, C-8, C-9, and C-16), and the correlations of H-17/H-22 and H-22/H-19 demonstrated the relative configurations at C-19 and C-22 as shown (Figure 3). Compound **3** was thus identified to be 19-*O*-acetyl-21,22-dihydro-22-methoxychaetoglobosin A.

Compound **4** was assigned the molecular formula C_36_H_44_N_2_O_7_ by HR-ESIMS analysis at *m*/*z* 617.3222 ([M + H]^+^, calcd. 617.3221), with 14 mass units (CH_2_) more than **3** indicative of a methylated analogue of the latter. Examination of the NMR data (in CDCl_3_, Table 1) for **4** confirmed this hypothesis, with resonances (*δ*_C_ 15.5, 64.5; *δ*_H_ 1.27 (t, *J* = 7.0 Hz), 3.56 (m), 3.67 (m)) for an ethoxy group replacing those for the 22-OMe in **3**, which was further supported by the diagnostic HMBC correlations from the ethoxy methylene protons to C-22 (Figure S26, Supplementary Materials). Careful inspection of the full 2D NMR data (Figures S24–S26, Supplementary Materials) secured the planar structure of **4** as shown. However, the relative configuration of **4** seemed to be different from that of **3**, as implied by the remarkable NMR variations around the C-22 chiral center. The most severe change occurred to the coupling pattern between H-22 and H_2_-21 (*J*_21,22_ = 5.8, 4.0 Hz in **4** vs. that (10.1, 1.7 Hz) in **3**), indicating a likely inverted C-22 configuration. Finally, the relative configuration of **4** was confirmed by interpretation of the NOESY data (Figure 3) to be consistent with that of **3** except at C-22, which was supported by the key correlation of H-8 with H-22. Compound **4** could be derived from **3** via a S_N_2 nucleophilic substitution of the ethoxy group, leading to the configurational inversion of C-22. Compound **4** was thereby characterized as 19-*O*-acetyl-21,22-dihydro-22-ethoxychaetoglobosin A.

Compound **5** was assigned the same molecular formula as **3** based on its HR-ESIMS ion at *m*/*z* 620.3334 ([M + NH_4_]^+^, calcd. 620.3330), suggestive of an isomer of the latter. Analysis of the NMR data (in CDCl_3_, Table 1) for **5** confirmed this hypothesis, with all its spectroscopic features being consistent with those in **3**. Further examination of the full 2D NMR data (Figures S31–S34, Supplementary Materials) demonstrated that compound **5** was a regio-isomer of **3**, with the methoxy group located at C-21 as evidenced by the HMBC correlation from the methoxy protons to C-21. The relative configurations of all chiral centers except for C-21 in **5** were assigned to be consistent with those of their counterparts in **3** by careful inspection of the NOESY data (Figure 3), and the C-21 configuration was established as drawn via the pivotal correlation of H-19/H-21. Compound **5** was hence identified to be 19-*O*-acetyl-21,22-dihydro-21-methoxychaetoglobosin A.

**Figure 5:**
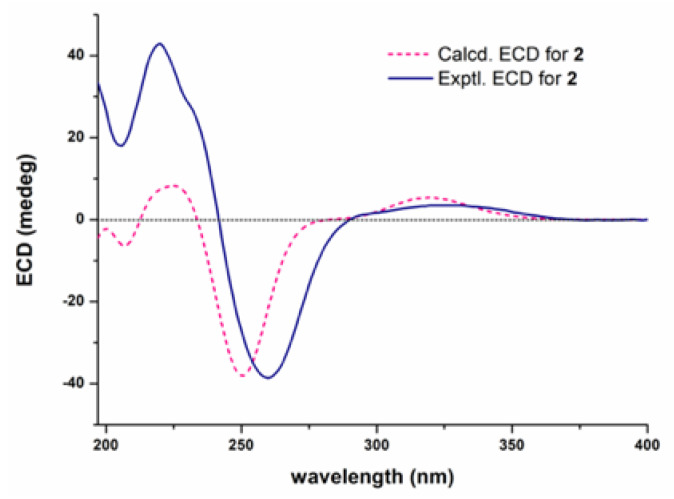
Experimental and calculated ECD spectra for **2**.

The absolute configuration of compound **2** was established by comparing its experimental ECD (electronic circular dichroism) spectrum with the calculated ones for its two enantiomers. As shown in Figure 5, the measured ECD curve of **2** showed a good match with the computed curve for the (3*S*,4*R*,5*S*,6*R*,7*S*,8*R*,9*R*,16*S*,20*S*)-isomer. Based on a biogenetic correlation with **2**, the absolute configurations of chaetoglobosins **3**–**5** were assigned as drawn.

Four known chaetoglobosins, namely chaetoglobosin F (**6**) [29], chaetoglobosin A (**7**) [29], penochalasin F (**8**) [30], and chaetoglobosin C (**9**) [29], were also isolated in the present work. These were identified by detailed spectroscopic analyses and comparison with the reported data in the literature.

### 3.2. Anti-Phytopathogenic Evaluation

The preliminary antifungal activity of the isolated chaetoglobosins against two pathogenic fungi was first evaluated at 10 μg/mL, and the results in Table S1 (Supplementary Materials) showed that these chaetoglobosins, except **1**, all exhibited various degrees of fungicidal effect. The EC_50_ values of the active chaetoglobosins toward *B. cinerea* were further measured, and the results are presented in Table 2. Most of these chaetoglobosins exhibited significant fungicidal activity against *B. cinerea* and were more potent than the commercial fungicides azoxystrobin and carbendazim, with EC_50_ values for **2**, **6**, **7**, and **9** of 2.19, 8.25, 0.40, and 5.83 μg/mL, respectively. The new compound **2** was chosen for the subsequent in-depth biological assessment against *B. cinerea*, given its sufficient sample supply and good fungicidal activity.

**Figure 6:**
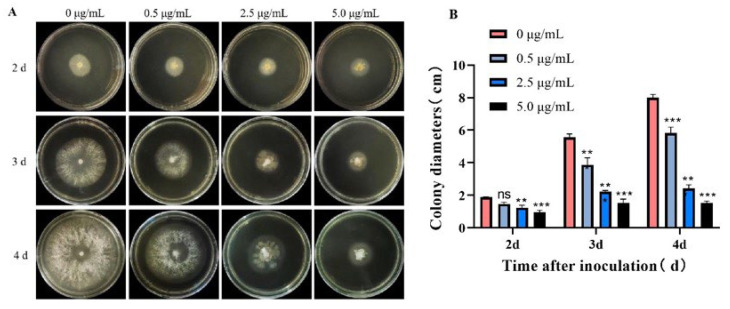
Antifungal evaluation of **2** against *B. cinerea*. (**A**,**B**) The morphology and colony diameter of *B. cinerea* on PDA plate after treatment; data are presented as the mean ± standard deviation. ns: not statistically significant; * *p* < 0.05, ** *p* < 0.01 and *** *p* < 0.001 compared with the control group (0 μg/mL).

The fungicidal effect of **2** on *B. cinerea* was further evaluated in a time and dose-dependent manner. As shown in Figure 6, compound **2** could effectively inhibit the growth of the pathogen at a concentration down to 0.5 μg/mL (a dose of about 1/5 of the EC_50_ value), and the inhibitory effect became more apparent as time went on. Even on the fourth day, the fungal colony diameter of the treatment group administrated with 5.0 μg/mL (about 2-fold of the EC_50_ value) **2** was still smaller than that of the control group on the second day. These observations demonstrate that compound **2** shows good suppressing activity toward *B. cinerea* and could be used as a protective agent against pathogenic infection.

**Figure 7:**
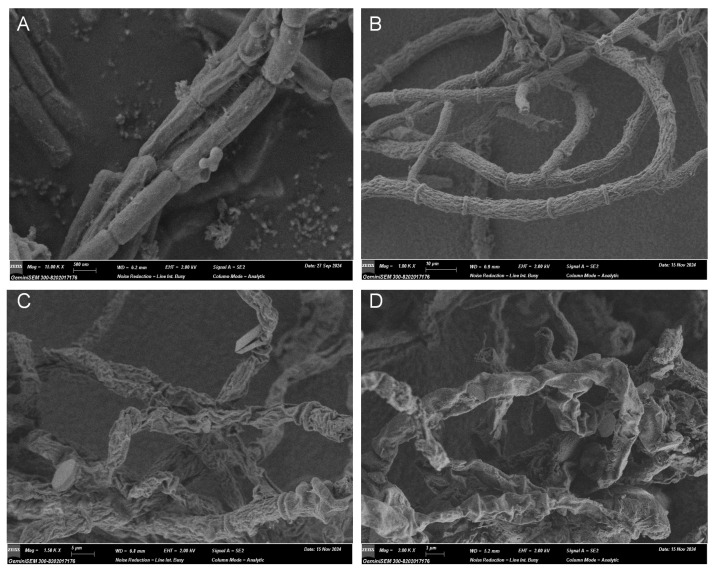
Representative pictures of the hypha morphology of *B. cinerea* observed under SEM. (**A**) Blank control with 1% DMSO (15.00 k×, 0.5 μm); (**B**) positive control group treated with azoxystrobin at 39.0 μg/mL (1.00 k×, 10 μm); (**C**) treated group with compound **2** at 2.5 μg/mL (1.50 k×, 5 μm); (**D**) treated with **2** at 5.0 μg/mL (2.00 k×, 3 μm).

Next, the morphological changes of *B. cinerea* upon the treatment of **2** were observed under a scanning electron microscope. As shown in Figure 7 (amplified images in supporting material Figure S46), the hyphae of *B. cinerea* in the control group (treated with only 1% DMSO) looked normal, and the columnar surface was relatively smooth, intact, and uniform, while in the azoxystrobin-treated group (positive control), the hyphae surface became rough with many creases. In contrast, in the two groups treated with 2.5 and 5.0 μg/mL of **2**, the morphology of the fungus was severely impaired with distorted and shrunken hyphae. These findings indicate that compound **2** could exert antifungal activity by disrupting the normal physiological function of fungal hyphae, with a much better effect that the reference drug azoxystrobin.

## 4. Conclusions

Currently, extensive studies have established the potent antitumor activities of chaetoglobosin derivatives across diverse cancer cell lines [20]. In our preliminary assays using the A549 and MDA-MB-231 cell lines, the isolated chaetoglobosins exhibited moderate activity (IC_50_ >20 μM) and did not surpass the efficacy of the reported derivatives. Given that our primary objective was to explore the antimicrobial activity of understudied chaetoglobosins, we prioritized the further investigation of their antimicrobial potential based on ecological relevance.

In this study, our chemical investigation into the culture fermentation of *Chaetomium* sp. UJN-EF006 derived from the leaves of *V. bracteatum* afforded nine chaetoglobosins including five previously undescribed ones (**1**–**5**). Most of these chaetoglobosins exhibited excellent antifungal activity against two phytopathogenic fungi. The new and major compound **2** was found to possess a strong fungicidal effect against *B. cinerea* with an EC_50_ value of 2.19 µg/mL, being much better than that (39.02 µg/mL) of the control drug azoxystrobin. A preliminary mechanistical study revealed that compound **2** could exert its antifungal activity by suppressing the normal growth and formation of fungal hyphae. To sum up, our current study demonstrates that *Chaetomium* sp. UJN-EF006 could be used as a biocontrol species, and its secondary metabolites could be developed into fungicides for the prevention and control of agricultural pathogens.

## Figures and Tables

**Table 1:** ^1^H and ^13^C NMR data of compounds **1**–**5**.

No.	1 ^a^		2 ^b^		3 ^b^		4 ^b^		5 ^b^	
	*δ*_C_	*δ*_H_, Multi. (*J* in Hz)	*δ*_C_	*δ*_H_, Multi. (*J* in Hz)	*δ*_C_	*δ*_H_, Multi. (*J* in Hz)	*δ*_C_	*δ*_H_, Multi. (*J* in Hz)	*δ*_C_	*δ*_H_, Multi. (*J* in Hz)
1	177.1		173.7		174.3		174.2		174.9	
2				5.80, brs		5.72, brs		5.69, brs		5.90, brs
3	60.5	3.33, dd (9.8, 5.5)	52.5	3.77, m	52.8	3.75, m	53.8	3.76, m	52.6	3.71, brdd (8.1, 7.1)
4	50.0	2.94, brs	46.5	3.30, dd (5.8, 2.4)	51.3	2.40, dd (5.5, 2.1)	50.2	2.45, brs	48.5	2.45, brd (6.3)
5	127.7		36.1	1.77, m	36.6	1.91, m	35.9	1.87, m	36.6	1.76, m
6	134.4		58.3		57.2		57.3		57.3	
7	70.0	3.81, brd (9.7)	62.8	2.82, d (5.5)	61.6	2.82, d (5.7)	61.4	2.77, d (5.4)	61.1	2.86, d (5.6)
8	52.6	2.13, dd (10.0, 9.7)	49.6	2.09, dd (10.2, 5.5)	49.6	2.59, dd (9.8, 5.7)	46.2	2.81, dd (9.7, 5.4)	47.3	2.38, dd (9.9, 5.6)
9	63.8		64.1		61.9		62.5		64.8	
10	42.0	2.66, dd (13.4, 5.5)	34.9	2.87, dd (14.4, 4.8)	34.4	2.84, d (7.3, 2H)	34.4	2.93, m (2H)	33.9	2.89, dd (14.1, 8.1)
		2.18, dd (13.4, 9.8)		2.61, dd (14.4, 8.6)						2.72, dd (14.1, 7.1)
11	17.4	1.28, brs	13.1	1.14, d (7.2)	13.1	1.10, d (7.3)	13.9	1.14, d (7.3)	12.4	0.78, d (7.2)
12	14.8	1.64, d (1.6)	20.0	1.23, s	19.8	1.22, s	20.3	1.25, s	19.4	1.12, s
13	129.4	6.28, ddd (15.3, 10.0, 2.0)	129.0	6.50, m (overlap)	128.7	6.17, ddd (15.1, 9.8, 1.9)	128.0	6.04, m	126.6	6.31, ddd (15.4, 9.9, 1.8)
14	136.1	5.23, ddd (15.3, 11.0, 2.9)	133.6	5.23, ddd (14.9, 11.1, 3.1)	133.7	5.21, ddd (15.1, 11.0, 3.6)	134.8	5.34, m	136.7	5.31, ddd (15.4, 10.8, 2.6)
15	42.2	2.48, m	41.7	2.41, m	41.8	2.28, m	41.3	2.31, m	40.1	2.36, m
		2.08, ddd (13.6, 12.1, 11.0)		2.19, ddd (14.2, 11.7, 11.1)		2.00, m		1.99, m		2.00, ddd (14.5, 11.8, 10.8)
16	34.6	2.82, m	33.9	2.69, m	32.3	2.47, m	33.1	2.60, m	33.3	2.68, m
17	150.5	6.31, brd (9.5)	152.1	6.56, brd (8.4)	142.3	5.66, dd (8.7, 1.5)	141.9	5.47, brd (10.2)	142.3	5.36, dd (10.5, 1.6)
18	137.0		133.8		128.4		126.8		128.7	
19	205.5		192.7		84.1	5.79, s	85.0	5.31, s	82.9	5.15, s
20	71.9	4.91, dd (7.8, 4.3)	76.2	6.38, d (10.7)	203.3		202.0		203.8	
21	32.0	1.93, m	136.8	6.50, dd (15.3, 10.7)	42.7	2.94, dd (15.5, 1.7)	40.5	3.62, dd, (18.0, 5.8)	79.8	4.67, dd (7.3, 2.7)
		1.85, m				2.65, dd (15.5, 10.1)		2.50, dd, (18.0, 4.4)		
22	38.2	3.03, dt (19.6, 5.7)	132.0	7.44, d (15.3)	80.8	4.77, dd (10.1, 1.7)	79.1	4.77, dd (5.8, 4.0)	43.0	3.41, dd (18.3, 2.7)
		2.91, ddd (19.6, 9.1, 5.4)								2.78, dd (18.3, 7.3)
23	211.2		194.1		206.2		205.1		204.6	
24	20.2	1.07, d (6.7)	20.9	1.06, d (6.9)	20.9	1.02, d (6.8)	21.2	1.02, d, (6.6)	21.0	1.02, d (6.5)
25	12.4	1.83, d (1.3)	11.7	1.75, brs	11.9	1.57, d (1.3)	11.7	1.55, d (1.3)	11.8	1.68, d (1.3)
1′	129.1			8.11, brs		8.13, brs		8.12, brs		8.11, d (2.3)
2′	131.4	6.93, d (8.4)	122.6	7.03, d (2.3)	122.69 ^c^	6.98, d (2.3)	122.68 ^d^	7.07, d (2.3)	122.5	7.08, d (2.3)
3′	116.4	6.74, d (8.4)	111.2		112.0		112.1		111.5	
3a′			127.1		127.0		127.0		127.1	
4′	157.4		118.5	7.52, brd (7.9)	118.5	7.51, brd, (7.9)	118.5	7.55, brd, (7.9)	118.5	7.57, brd (7.9)
5′	116.4	6.74, d (8.4)	120.1	7.16, m	120.1	7.15, m	120.1	7.17, m	120.0	7.15, m
6′	131.4	6.93, d (8.4)	123.2	7.22, m	122.74 ^c^	7.23, m	122.71 ^d^	7.24, m	123.4	7.22, m
7′			111.6	7.37, brd (8.1)	111.7	7.40, brd (8.1)	111.7	7.41, brd, (8.1)	111.6	7.39, brd (8.1)
7a′			136.5		136.6		136.6		136.5	
OAc			19.8	2.21, s	21.2	2.18, s	170.5		20.6	2.12, s
			169.9		170.2		20.8	2.13, s	171.2	
OMe(Et)					59.0	3.48	64.5	3.56, m	58.9	3.45, s
								3.67, m		
							15.5	1.27, t (7.0)		

^a^ Measured in CD_3_OD, ^b^ measured in CDCl_3_; ^b^ Measured in CD_3_Cl; ^c,d^ Interchangeable assignments.

**Table 2:** Toxicity regression equations and EC_50_ values of compounds against *B. cinerea*.

Compds.	EC_50_ (μg/mL)	95% CI ^a^	Regression Equation *y* = *ax* + b	Correlation Coefficient (*R*^2^)
**2**	2.19	1.1–5.40	*y* = 0.103*x* + 0.274	0.938
**3**	22.96	5.3–32.2	*y* = 0.015*x* + 0.165	0.963
**4**	19.32	2.9–35.1	*y* = 0.009*x* + 0.328	0.952
**5**	26.45	8.7–31.3	*y* = 0.007*x* + 0.454	0.979
**6**	8.25	5.1–34.0	*y* = 0.019*x* + 0.341	0.956
**7**	0.40	0.4–1.70	*y* = 0.203*x* + 0.418	0.957
**8**	18.17	7.4–52.6	*y* = 0.012*x* + 0.287	0.920
**9**	5.83	1.7–27.1	*y* = 0.019*x* + 0.392	0.942
Carbendazim	70.11	1.0–97.1	*y* = 0.003*x* + 0.304	0.998
Azoxystrobin	39.02	0.8–80.9	*y* = 0.004*x* + 0.328	0.971

^a^ Confidence interval.

## Data Availability

The data that support the findings of this study are available in the Supplementary Materials attached to this article.

## Supplementary Materials

The following supporting information can be downloaded at: https://www.mdpi.com/article/10.3390/jof11070511/s1, Table S1: Fungicidal activities of isolated compounds at 10 μg/mL; Figure S1: ^1^H NMR (600 MHz, CD_3_OD) spectrum of **1**; Figure S2: ^13^C and DEPT NMR (150 MHz, CD_3_OD) spectra of **1**; Figures S3–S6: 2D NMR spectra of **1**; Figure S7: The (+)-HR-ESIMS spectrum of **1**; Figure S8: ^1^H NMR (600 MHz, CDCl_3_) spectrum of **2**; Figure S9: ^13^C and DEPT NMR (150 MHz, CDCl_3_) spectra of **2**; Figures S10–S13: 2D NMR spectra of **2**; Figure S14: The (+)-HR-ESIMS spectrum of **2**; Figure S15: ^1^H NMR (600 MHz, CDCl_3_) spectrum of **3**; Figure S16: ^13^C and DEPT NMR (150 MHz, CDCl_3_) spectra of **3**; Figures S17–S20: 2D NMR spectra of **3**; Figure S21: The (+)-HR-ESIMS spectrum of **3**; Figure S22: ^1^H NMR (600 MHz, CDCl_3_) spectrum of **4**; Figure S23: ^13^C and DEPT NMR (150 MHz, CDCl_3_) spectra of **4**; Figures S24–S27: 2D NMR spectra of **4**; Figure S28: The (+)-HR-ESIMS spectrum of **4**; Figure S29: ^1^H NMR (600 MHz, CDCl_3_) spectrum of **5**; Figure S30: ^13^C and DEPT NMR (150 MHz, CDCl_3_) spectra of **5**; Figures S31–S34: 2D NMR spectra of **5**; Figure S35: The (+)-HR-ESIMS spectrum of **5**; Figure S36: ^1^H NMR (600 MHz, CDCl_3_) spectrum of **6**; Figure S37: ^13^C and DEPT NMR (150 MHz, CDCl_3_) spectra of **6**; Figure S38: ^1^H NMR (600 MHz, CDCl_3_) spectrum of **7**; Figure S39: ^13^C and DEPT NMR (150 MHz, CDCl_3_) spectra of **7**; Figure S40: ^1^H NMR (600 MHz, CDCl_3_) spectrum of **8**; Figure S41: ^13^C and DEPT NMR (150 MHz, CDCl_3_) spectra of **8**; Figure S42: ^1^H NMR (600 MHz, CDCl_3_) spectrum of **9**; Figure S43: ^13^C and DEPT NMR (150 MHz, CDCl_3_) spectra of **9**; Figure S44: Schematic Diagram of Compound Separation Process; Figure S45: ITS sequencing information of *Chaetomium globosum* and Phylogenetic tree analysis; Figure S46: The amplified representative pictures of the hypha morphology of *B. cinerea* observed under SEM.
