# Supplementary material for: New Chaetoglobosins with Fungicidal Activity from Chaetomium sp. UJN-EF006 Endophytic in Vaccinium bracteatum
Source: J Fungi (Basel). 2025 Jul 7;11(7):511. doi: 10.3390/jof11070511 (PMC12295373; doi:10.3390/jof11070511)
Supplement: Supplementary file 1 [file jof-11-00511-s001.zip › jof-3678177-supplementary.pdf]

***Supplementary materials* for**

New chaetoglobosins with fungicidal activity from *Chaetomium* sp. UJN-EF006 endophytic in *Vaccinium bracteatum*

Luo-Jing Wang[1,#], Zong-Yan Ma[1,#], Xin-Ling Wang[1], Kai-Le Wang[1], Tong Zhang[1], Rui-Ying Han[1], Jun-Jiang Li[1], Jie Bao[1,*], Yin-Yin Wang[1,2,*], Hua Zhang[1,*]

[1] *School of Biological Science and Technology, University of Jinan, Jinan 250022, China*

[2] *School of Chemistry and Chemical Engineering, University of Jinan, Jinan 250022, China*

[#] These authors contributed equally to this work

* Corresponding authors

Email addresses: yinyinwang0530@163.com (Y.-Y. W.); bio_baoj@ujn.edu.cn (J. B.); bio_zhangh@ujn.edu.cn (H. Z.)

List of Contents

Table S1. Fungicidal activities of isolated compounds at 10 μg/mL.

Figure S1. The ^{1}H NMR spectrum of **1** (CD_3OD)

Figure S2. The ^{13}C and DEPT NMR spectra of **1** (CD_3OD)

Figure S3. The ^{1}H-^{1}H COSY spectrum of **1** (CD_3OD)

Figure S4. The HSQC spectrum of **1** in (CD_3OD)

Figure S5. The HMBC spectrum of **1** (CD_3OD)

Figure S6. The NOESY spectrum of **1** (CD_3OD)

Figure S7. The (+)-HR-ESIMS spectrum of **1**

Figure S8. The ^{1}H NMR spectrum of **2** ($CDCl_3$)

Figure S9. The ^{13}C and DEPT NMR spectra of **2** ($CDCl_3$)

Figure S10. The ^{1}H-^{1}H COSY spectrum of **2** ($CDCl_3$)

Figure S11. The HSQC spectrum of **2** ($CDCl_3$)

Figure S12. The HMBC spectrum of **2** ($CDCl_3$)

Figure S13. The NOESY spectrum of **2** ($CDCl_3$)

Figure S14. The (+)-HR-ESIMS spectrum of **2**

Figure S15. The ^{1}H NMR spectrum of **3** ($CDCl_3$)

Figure S16. The ^{13}C and DEPT NMR spectra of **3** ($CDCl_3$)

Figure S17. The ^{1}H-^{1}H COSY spectrum of **3** ($CDCl_3$)

Figure S18. The HSQC spectrum of **3** ($CDCl_3$)

Figure S19. The HMBC spectrum of **3** ($CDCl_3$)

Figure S20. The NOESY spectrum of **3** ($CDCl_3$)

Figure S21. The (+)-HR-ESIMS spectrum of **3**

Figure S22. The ^{1}H NMR spectrum of **4** ($CDCl_3$)

Figure S23. The ^{13}C and DEPT NMR spectra of **4** ($CDCl_3$)

Figure S24. The ^{1}H-^{1}H COSY spectrum of **4** ($CDCl_3$)

Figure S25. The HSQC spectrum of **4** ($CDCl_3$)

Figure S26. The HMBC spectrum of **4** ($CDCl_3$)

Figure S27. The NOESY spectrum of **4** ($CDCl_3$)

Figure S28. The (+)-HR-ESIMS spectrum of **4**

Figure S29. The ^{1}H NMR spectrum of **5** ($CDCl_3$)

Figure S30. The ^{13}C and DEPT NMR spectra of **5** ($CDCl_3$)

Figure S31. The ^{1}H-^{1}H COSY spectrum of **5** ($CDCl_3$)

Figure S32. The HSQC spectrum of **5** ($CDCl_3$)

Figure S33. The HMBC spectrum of **5** ($CDCl_3$)

Figure S34. The NOESY spectrum of **5** ($CDCl_3$)

Figure S35. The (+)-HR-ESIMS spectrum of **5**

Figure S36. The ^{1}H NMR spectrum of **6** ($CDCl_3$)

Figure S37. The ^{13}C and DEPT NMR spectra of **6** ($CDCl_3$)

Figure S38. The ^{1}H NMR spectrum of **7** ($CDCl_3$)

Figure S39. The ^{13}C and DEPT NMR spectra of **7** ($CDCl_3$)

Figure S40. The ^{1}H NMR spectrum of **8** ($CDCl_3$)

Figure S41. The ^{13}C and DEPT NMR spectra of **8** ($CDCl_3$)

Figure S42. The ^{1}H NMR spectrum of **9** ($CDCl_3$)

Figure S43. The ^{13}C and DEPT NMR spectra of **9** ($CDCl_3$)

Figure S44. Schematic Diagram of Compound Separation Process.

Figure S45. ITS sequencing information of *Chaetomium globosum* and Phylogenetic tree analysis.

Figure S46. The amplified representative pictures of the hypha morphology of *B. cinerea* observed under SEM.

Table S1. Fungicidal activities (Inhibition rate %) of isolated compounds at 10 μg/mL.

Compds.	*B. cinerea*	*S. sclerotiorum*
**1**	0	0
**2**	64.42 ± 1.03	32.44 ± 2.69
**3**	35.94 ± 0.21	5.12 ± 0.14
**4**	36.41 ± 1.43	1.02 ± 2.12
**5**	39.05 ± 2.07	26.94 ± 3.24
**6**	58.60 ± 2.19	0
**7**	86.54 ± 0.96	60.0 ±1.0
**8**	26.60 ± 0.55	4.22 ± 3.85
**9**	60.25 ± 4.84	14.0 ± 0.50
azoxystrobin	23.43 ± 0.11	57.70 ± 0.81

Figure S1. The ^{1}H NMR spectrum of **1** (CD_3OD).

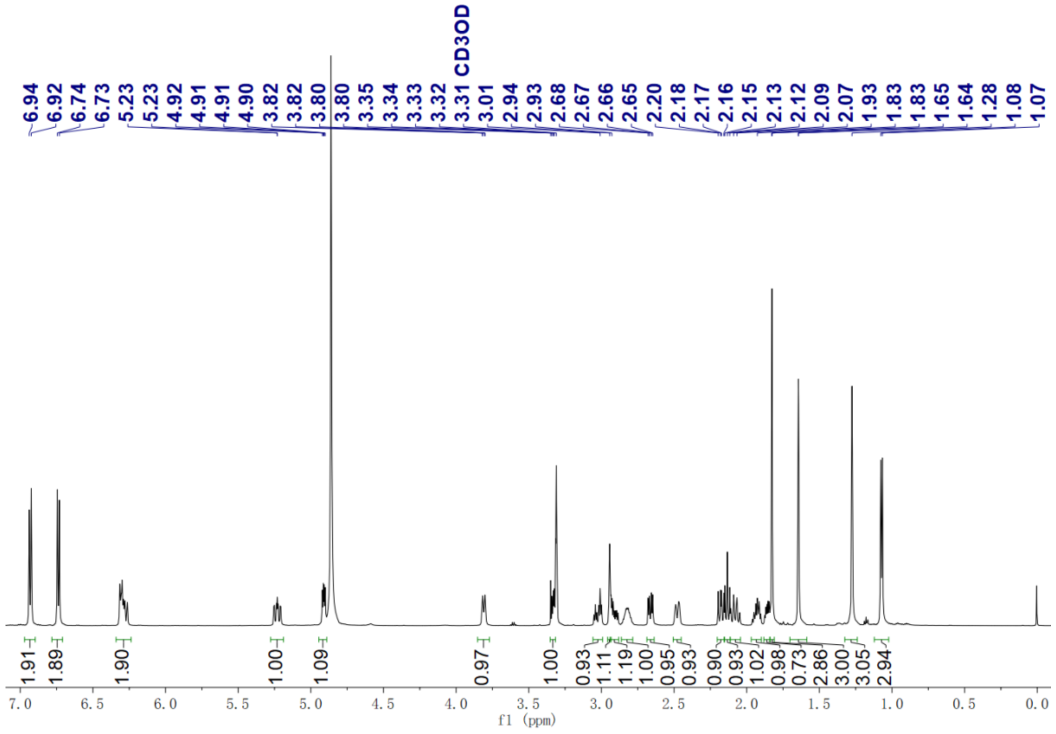

Figure S2. The ^{13}C and DEPT NMR spectra of **1** (CD_3OD).

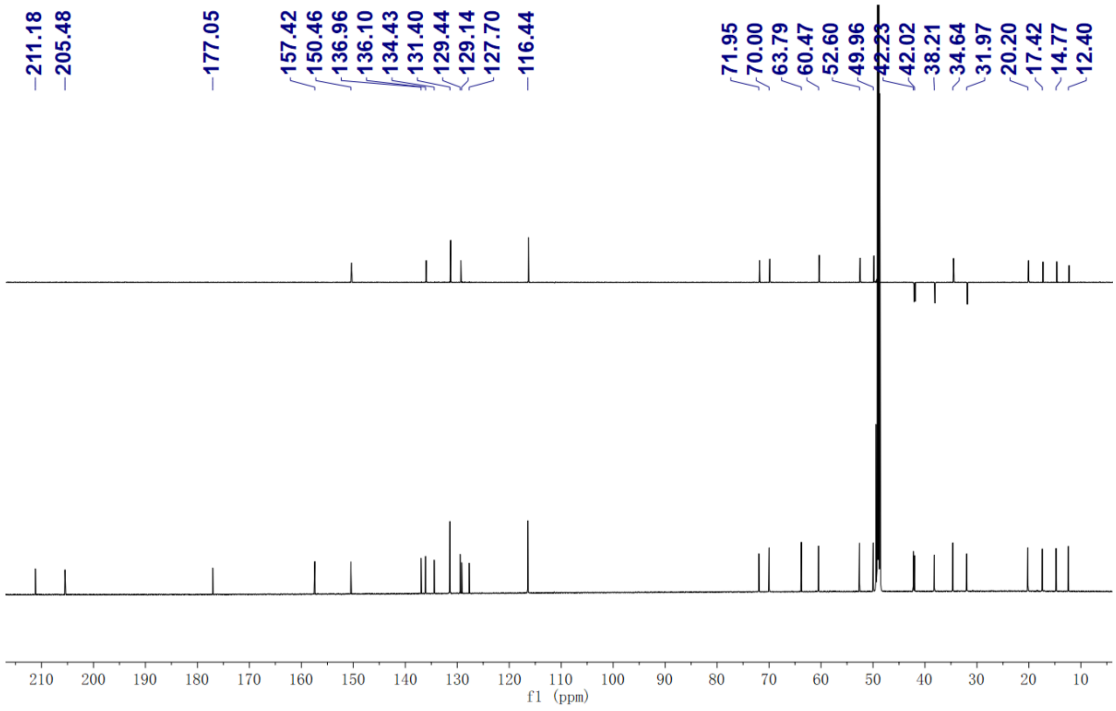

Figure S3. The ^{1}H-^{1}H COSY spectrum of **1** (CD_3OD)

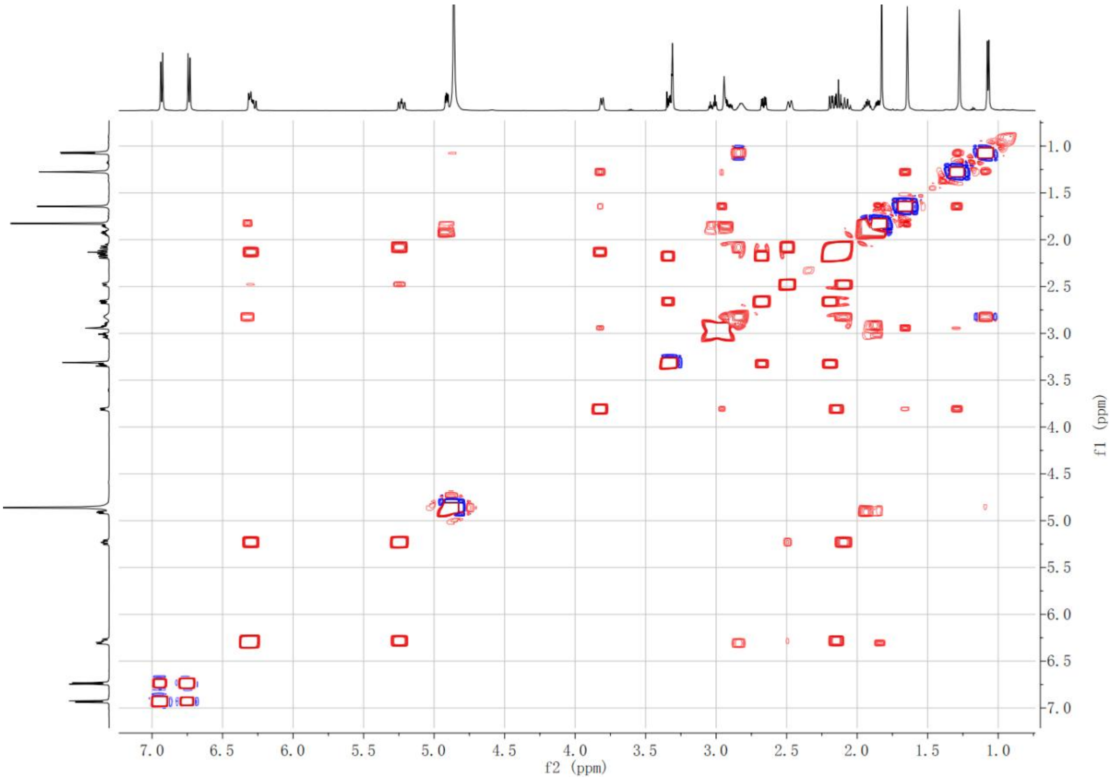

Figure S4. The HSQC spectrum of **1** in (CD_3OD)

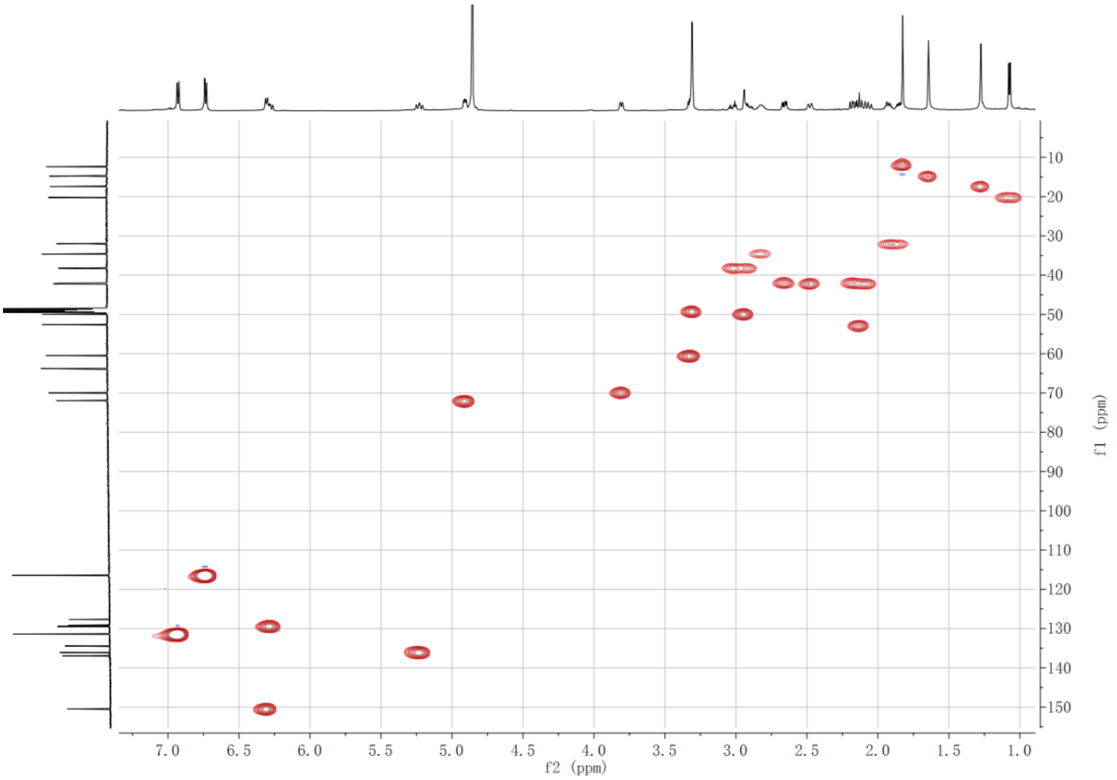

Figure S5. The HMBC spectrum of **1** (CD_3OD)

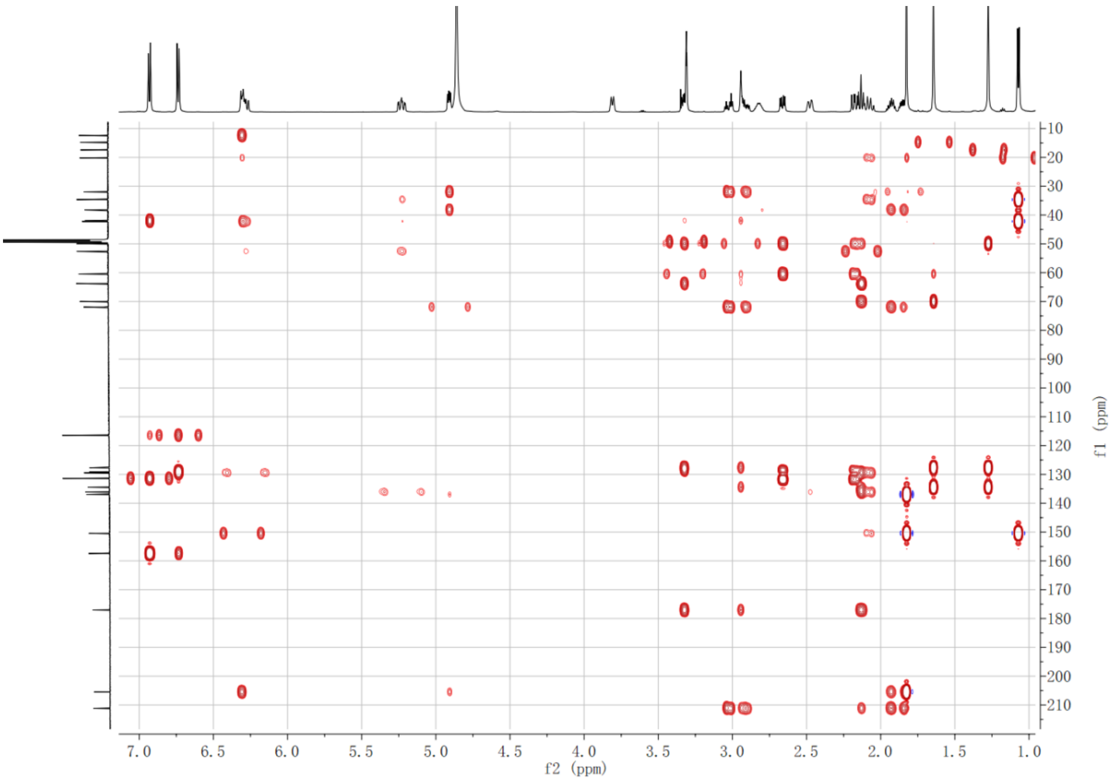

Figure S6. The NOESY spectrum of **1** (CD_3OD)

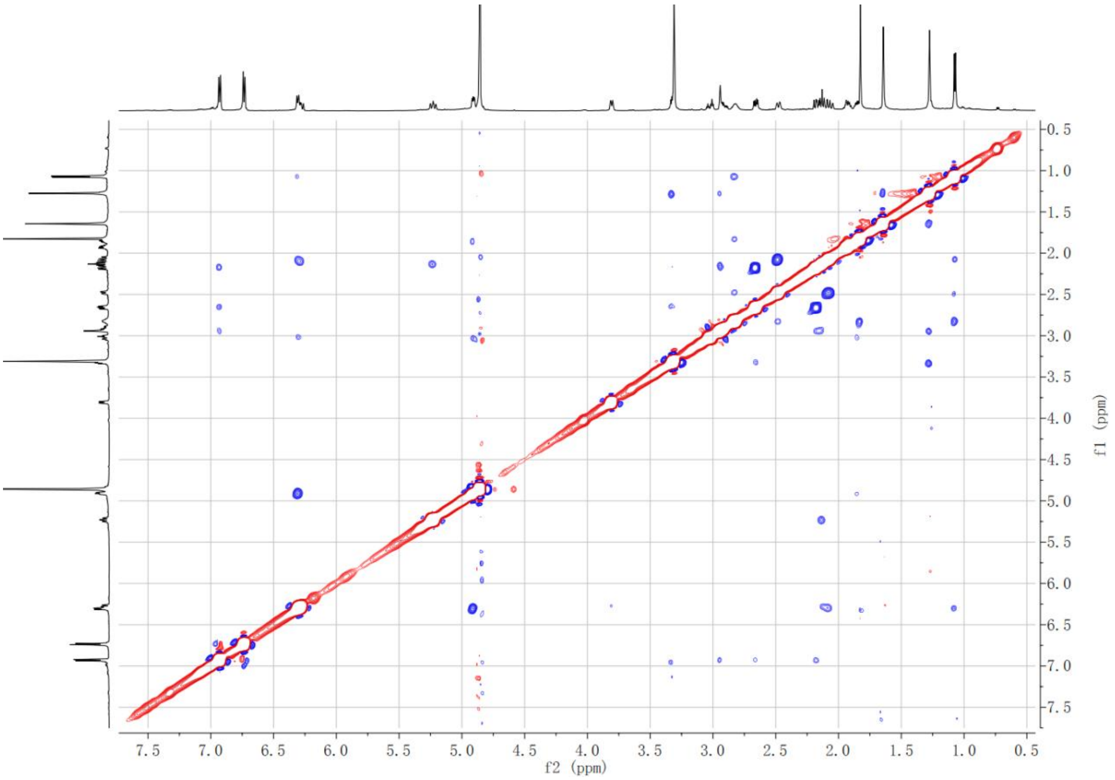

Figure S7. The (+)-HR-ESIMS spectrum of **1**.

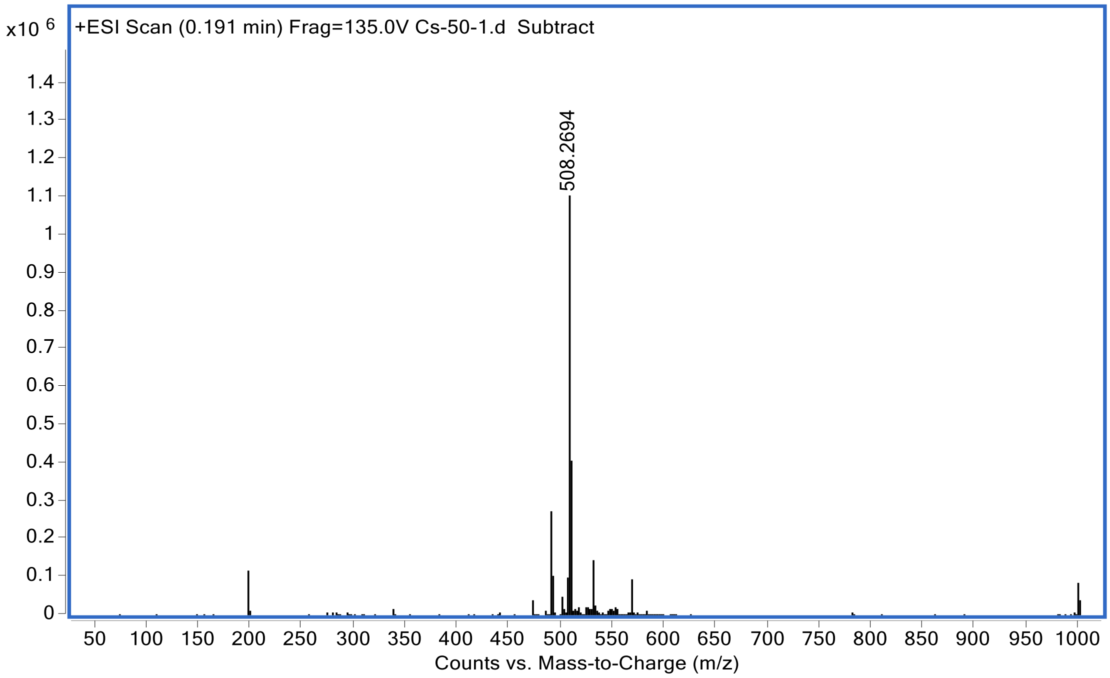

Figure S8. The ^{1}H NMR spectrum of **2** ($CDCl_3$).

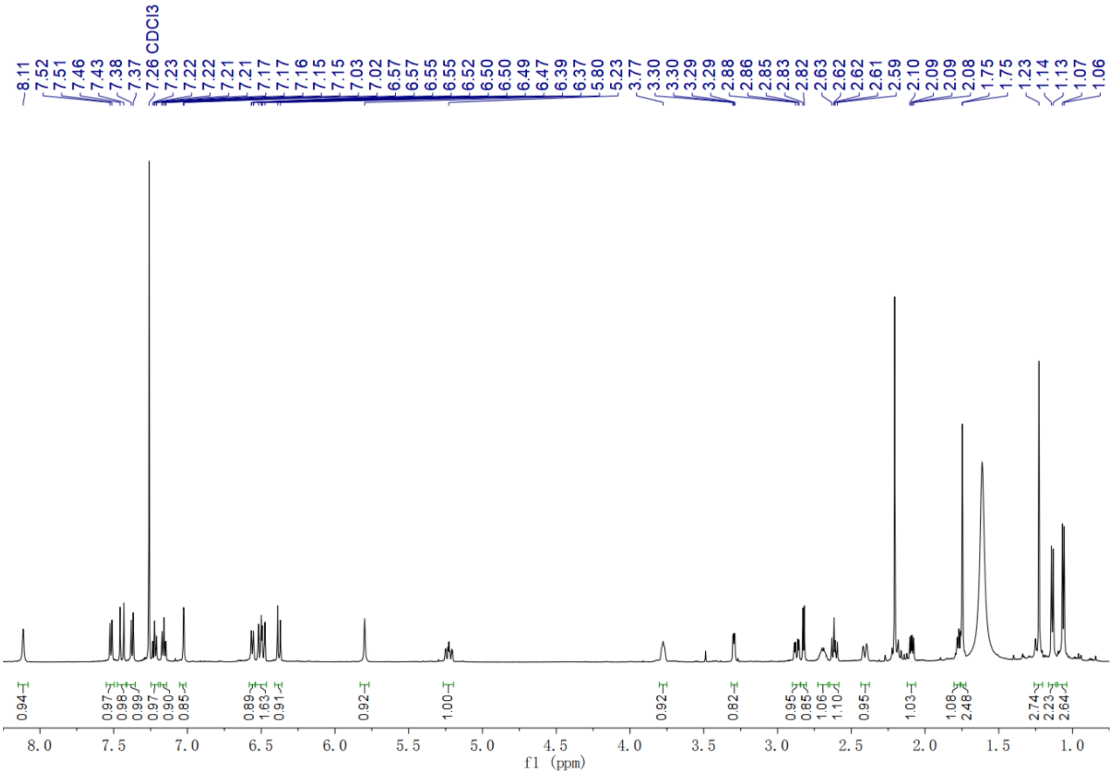

Figure S9. The ^{13}C and DEPT NMR spectra of **2** ($CDCl_3$).

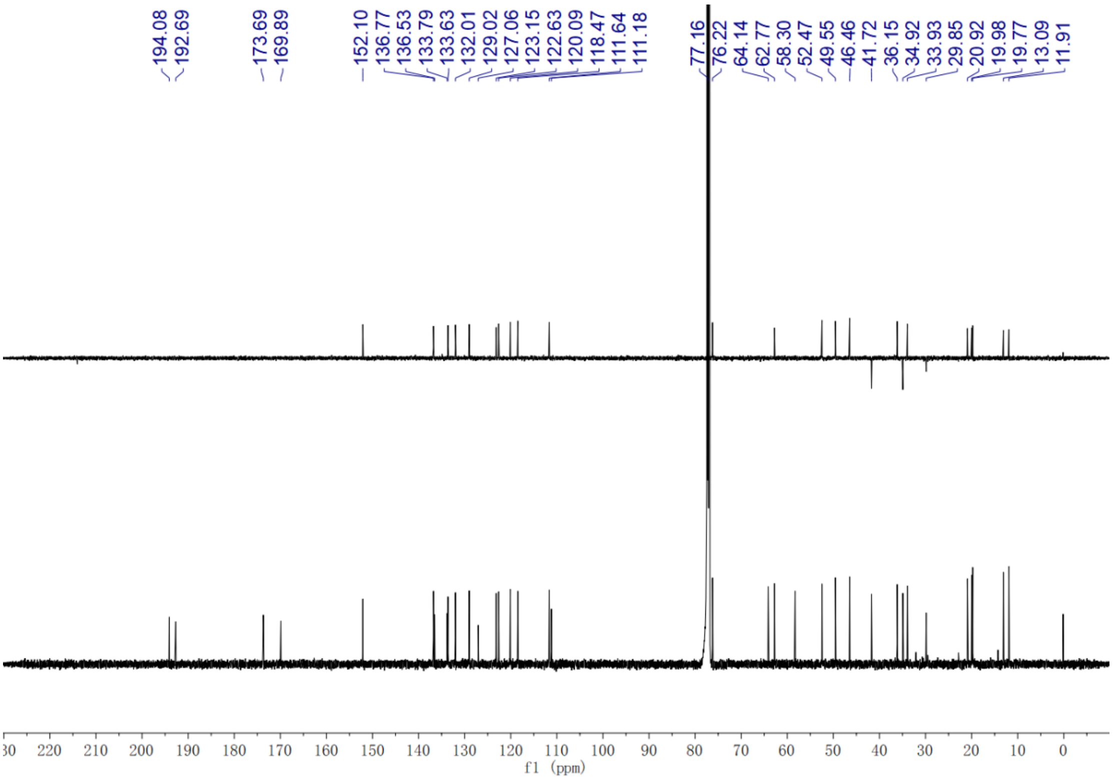

Figure S10. The ^{1}H-^{1}H COSY spectrum of **2** ($CDCl_3$).

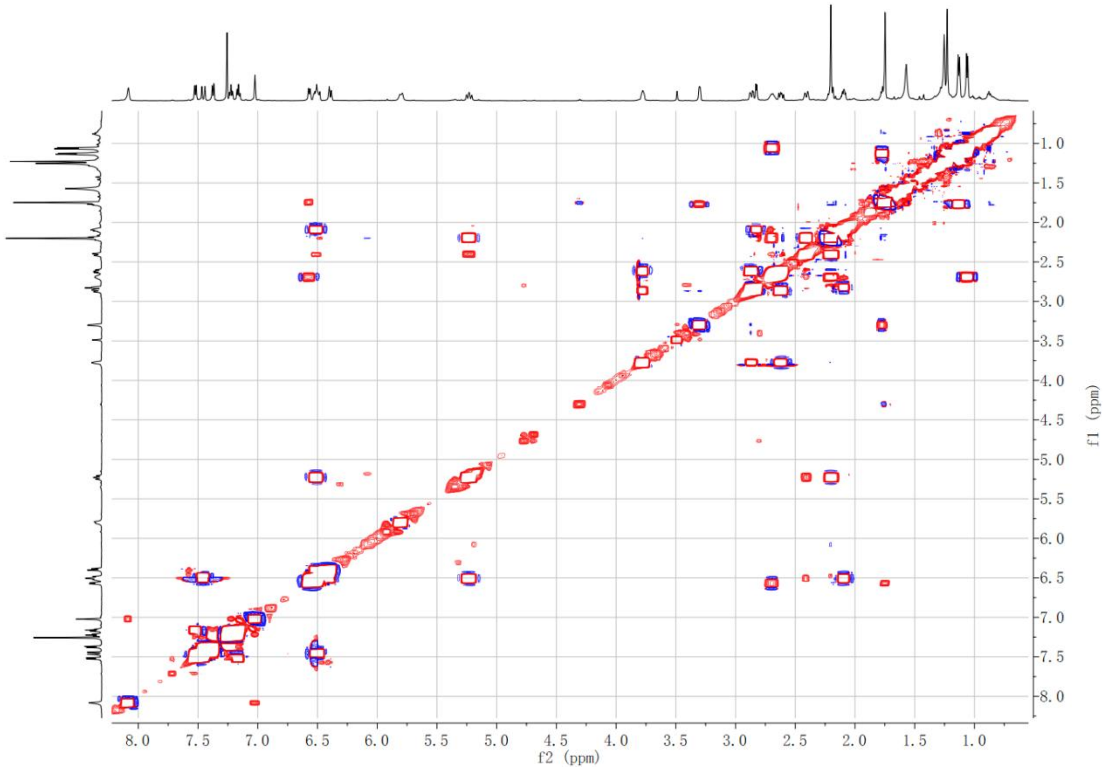

Figure S11. The HSQC spectrum of **2** ($CDCl_3$).

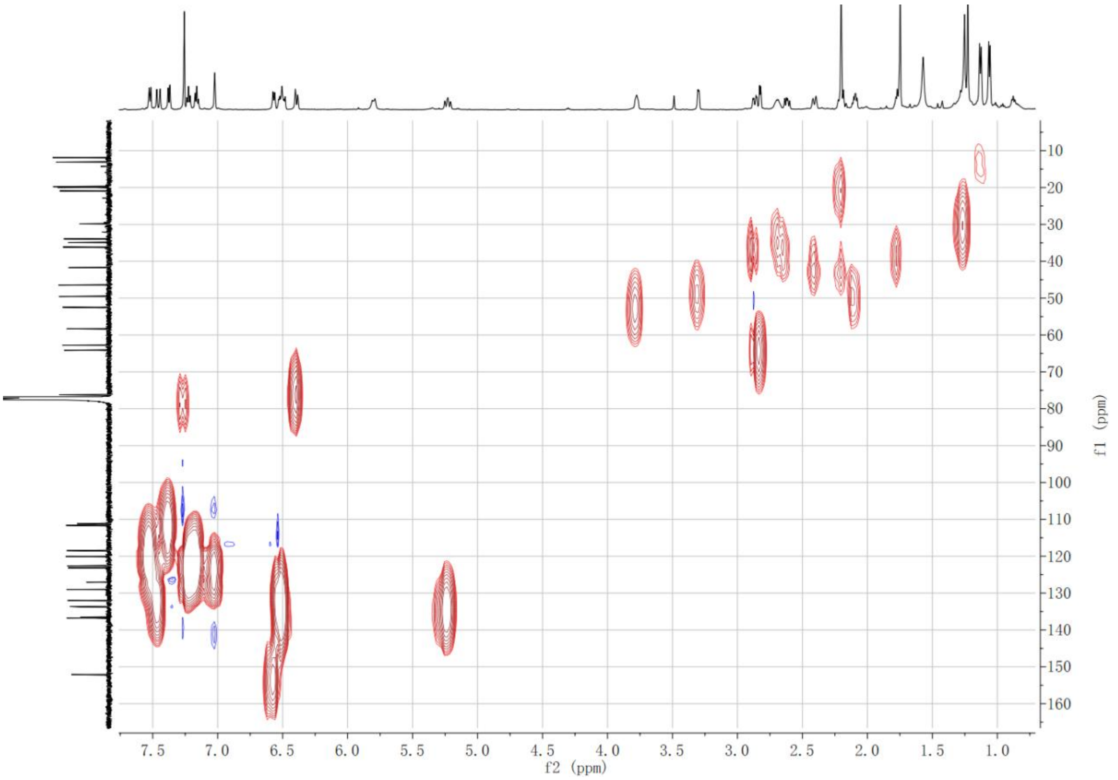

Figure S12. The HMBC spectrum of **2** ($CDCl_3$).

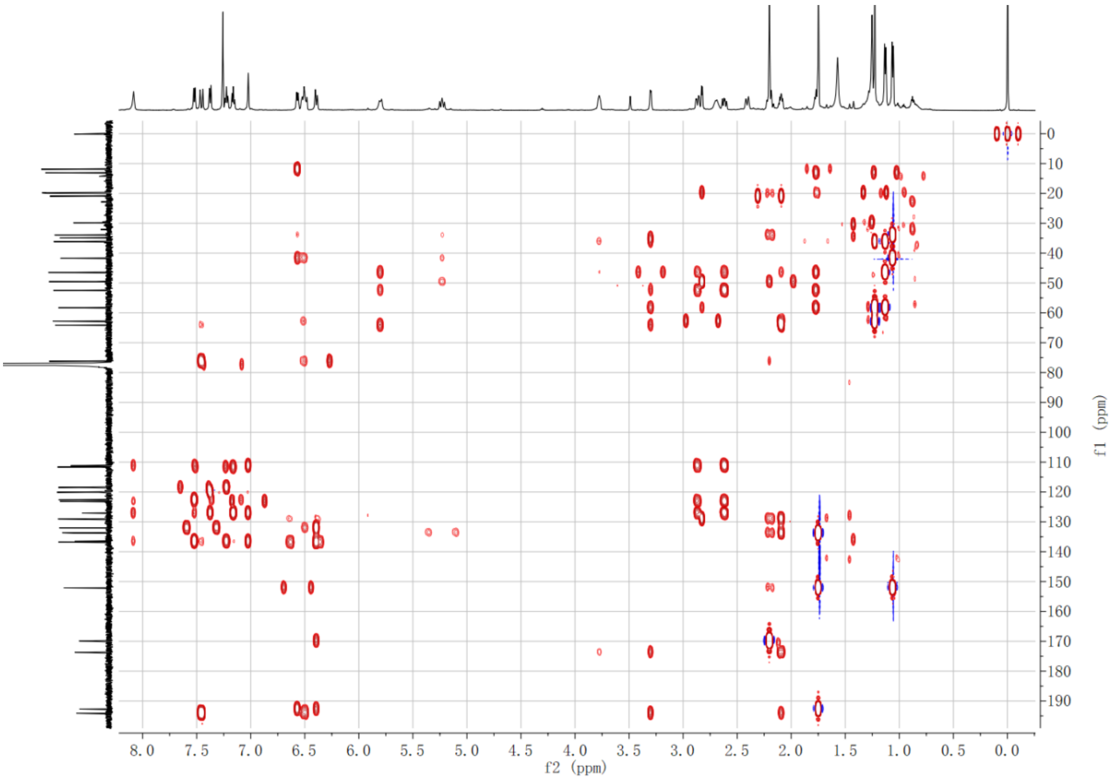

Figure S13. The NOESY spectrum of **2** ($CDCl_3$).

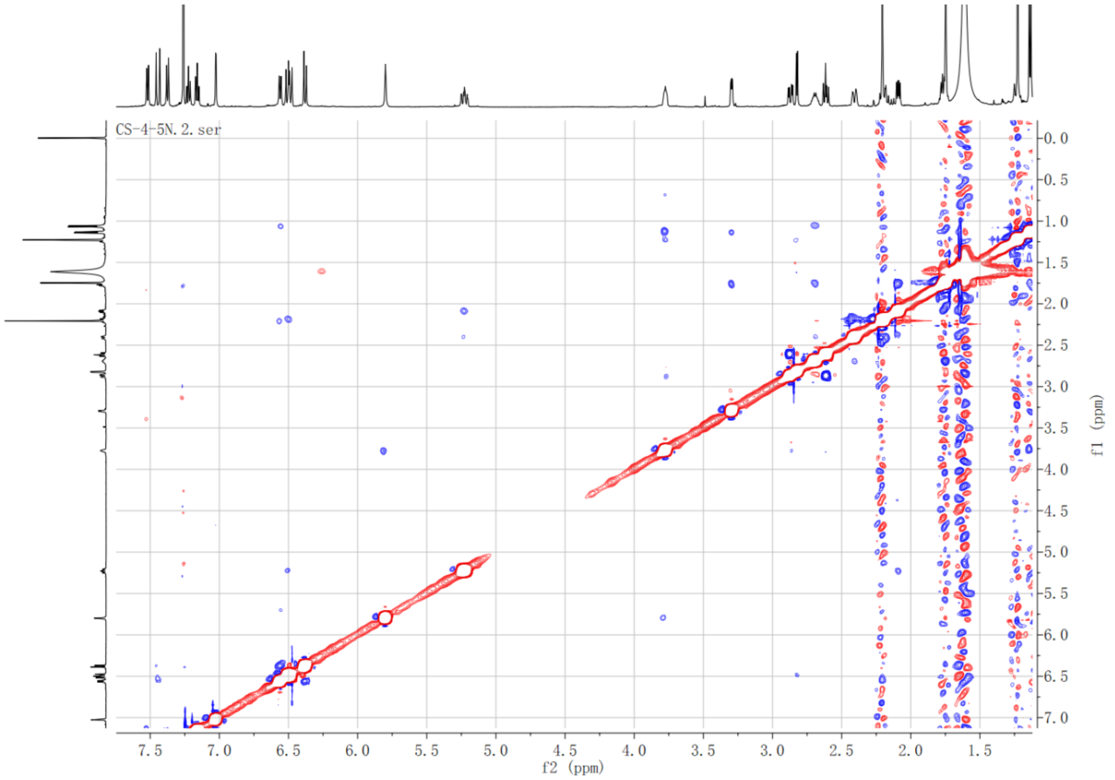

Figure S14. The (+)-HR-ESIMS spectrum of **2**.

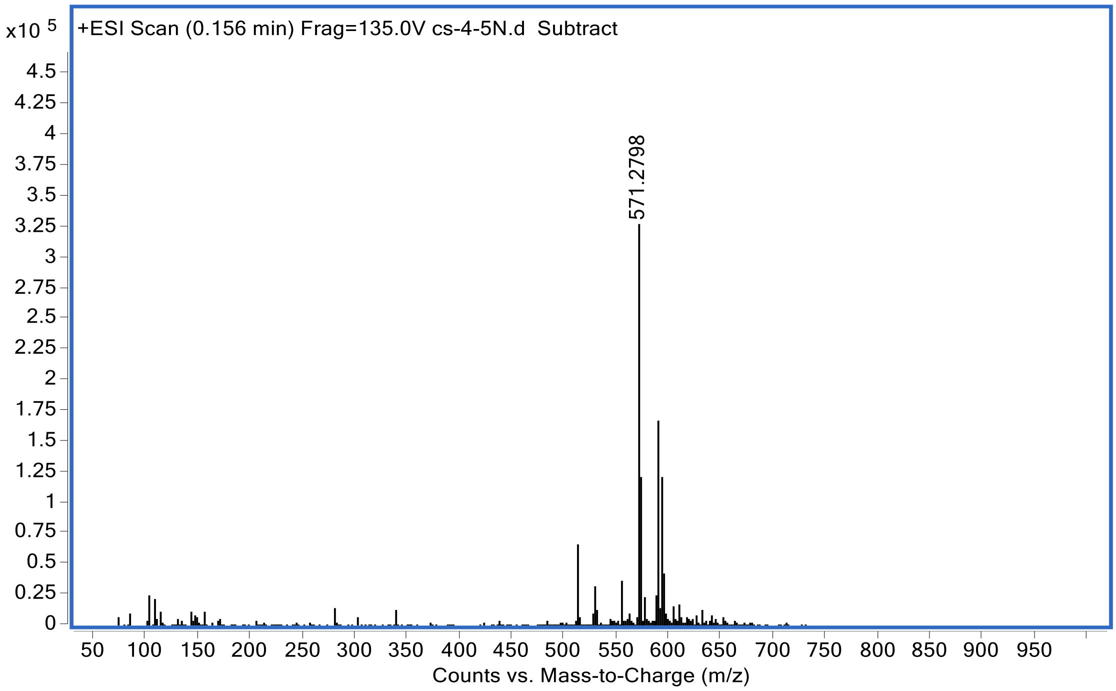

Figure S15. The ^{1}H NMR spectrum of **3** ($CDCl_3$).

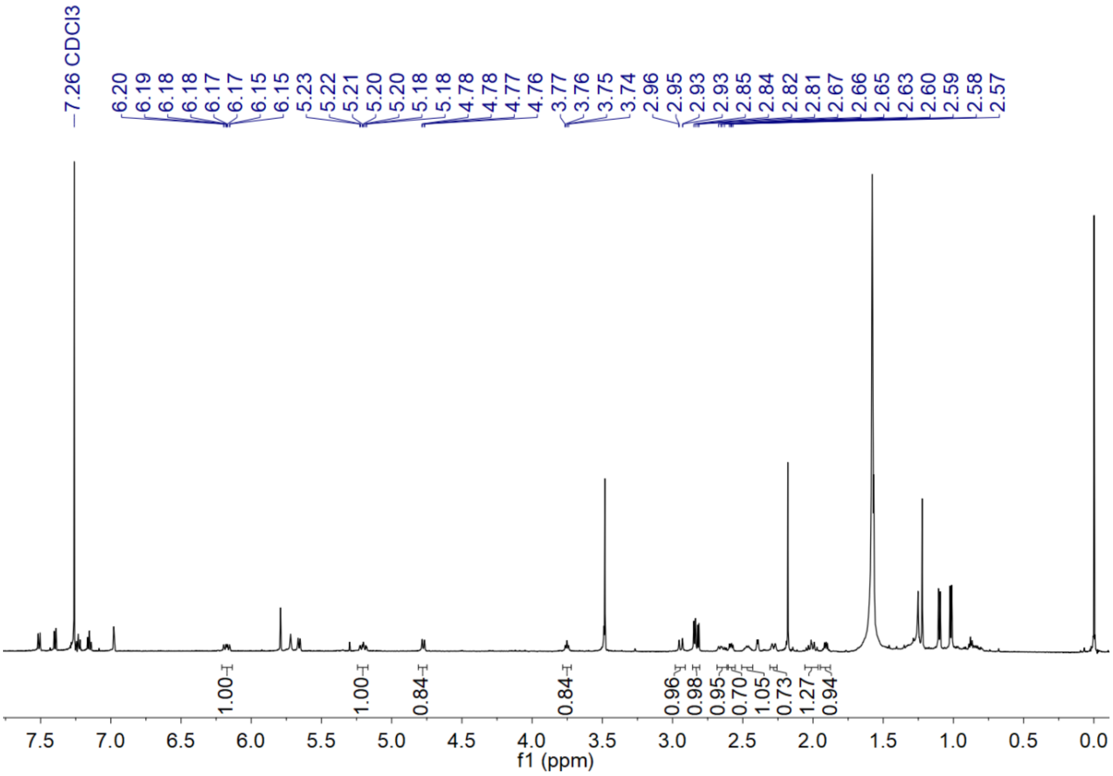

Figure S16. The ^{13}C and DEPT NMR spectra of **3** ($CDCl_3$).

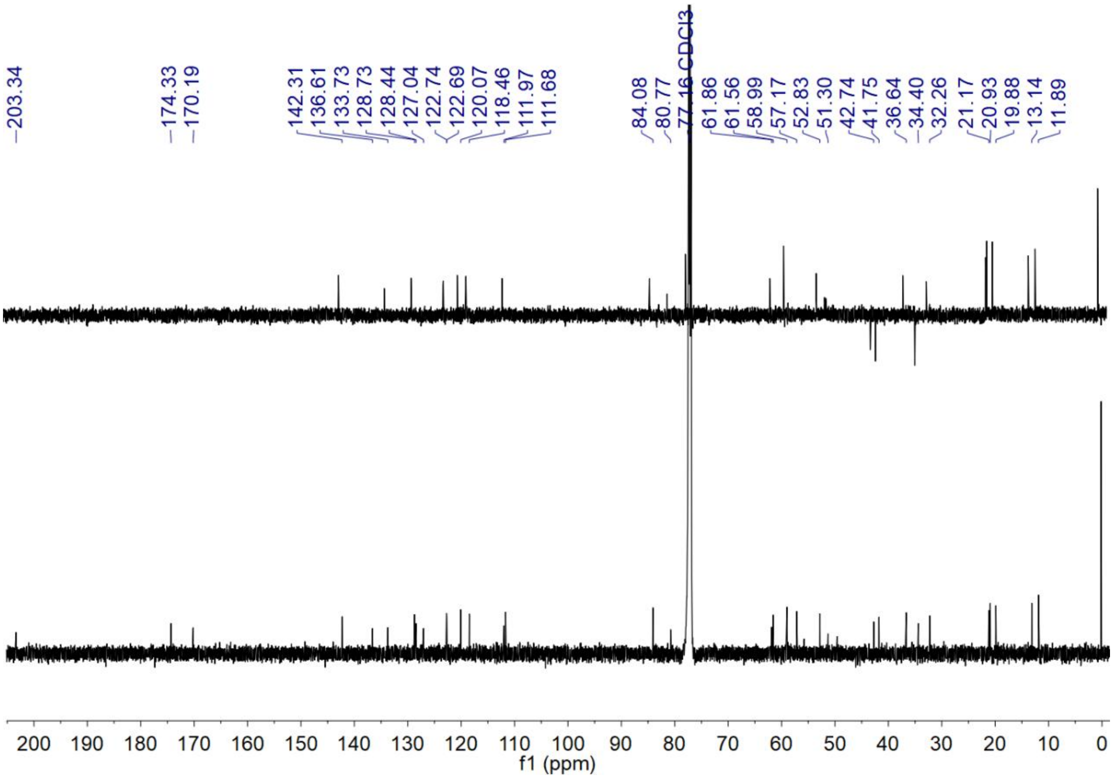

Figure S17. The ^{1}H-^{1}H COSY spectrum of **3** ($CDCl_3$).

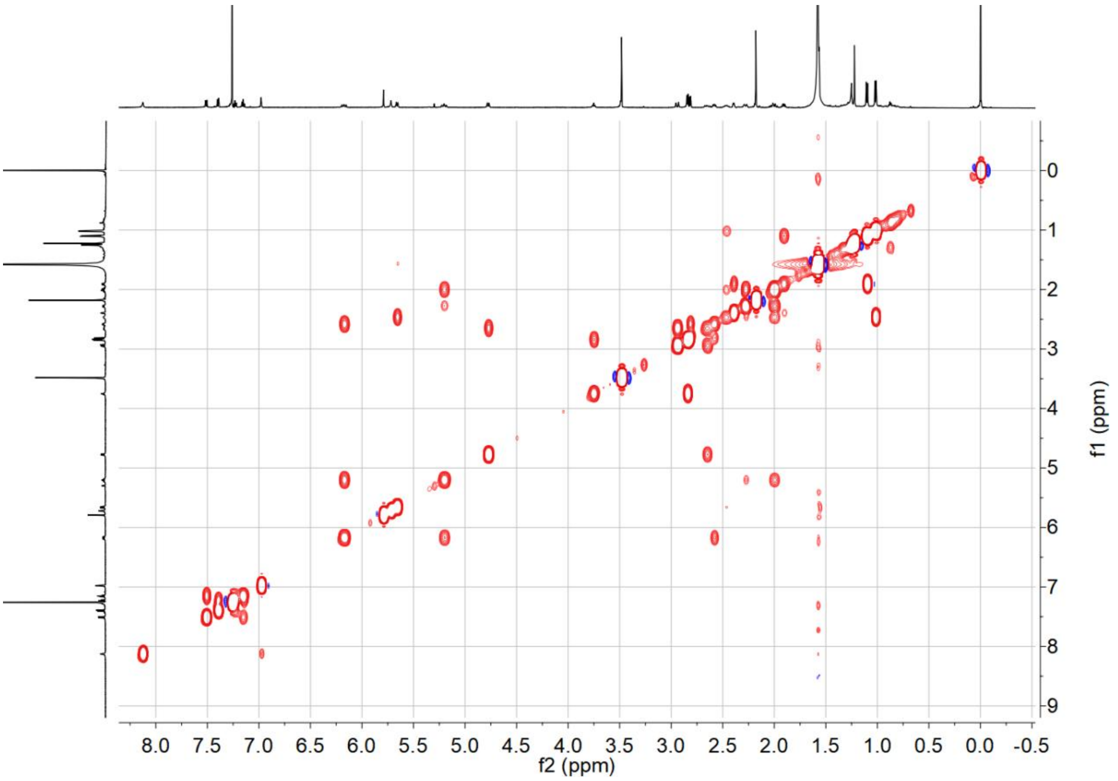

Figure S18. The HSQC spectrum of **3** ($CDCl_3$).

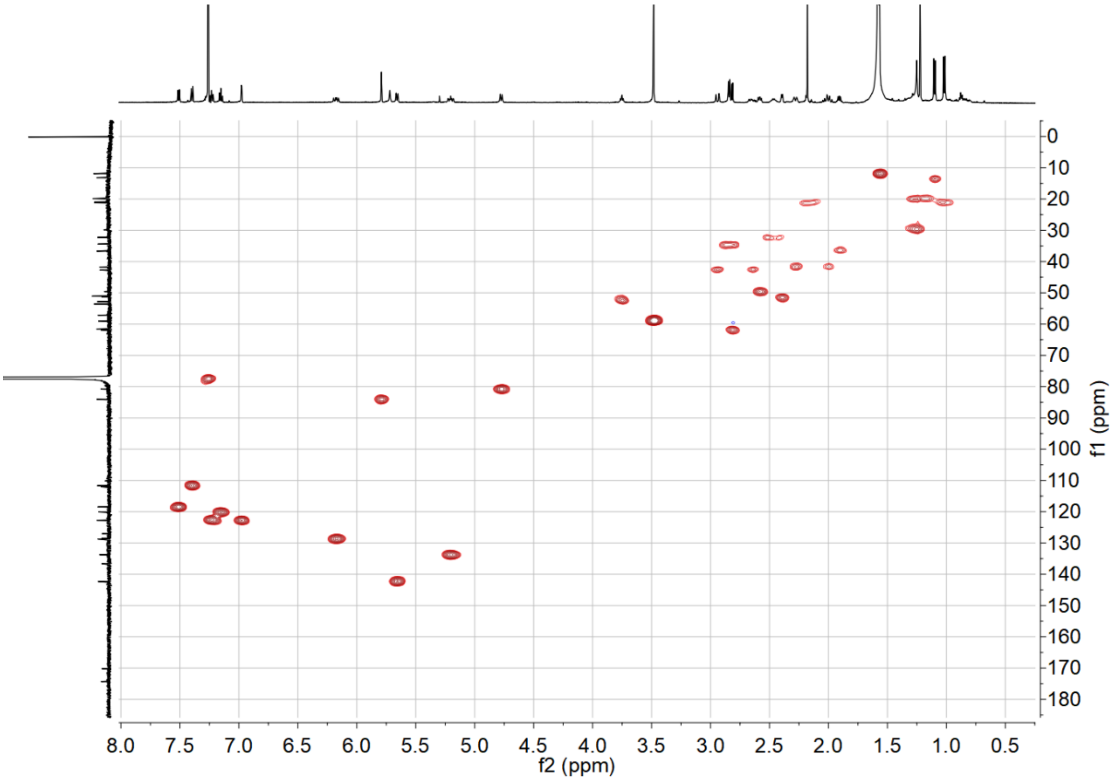

Figure S19. The HMBC spectrum of **3** ($CDCl_3$).

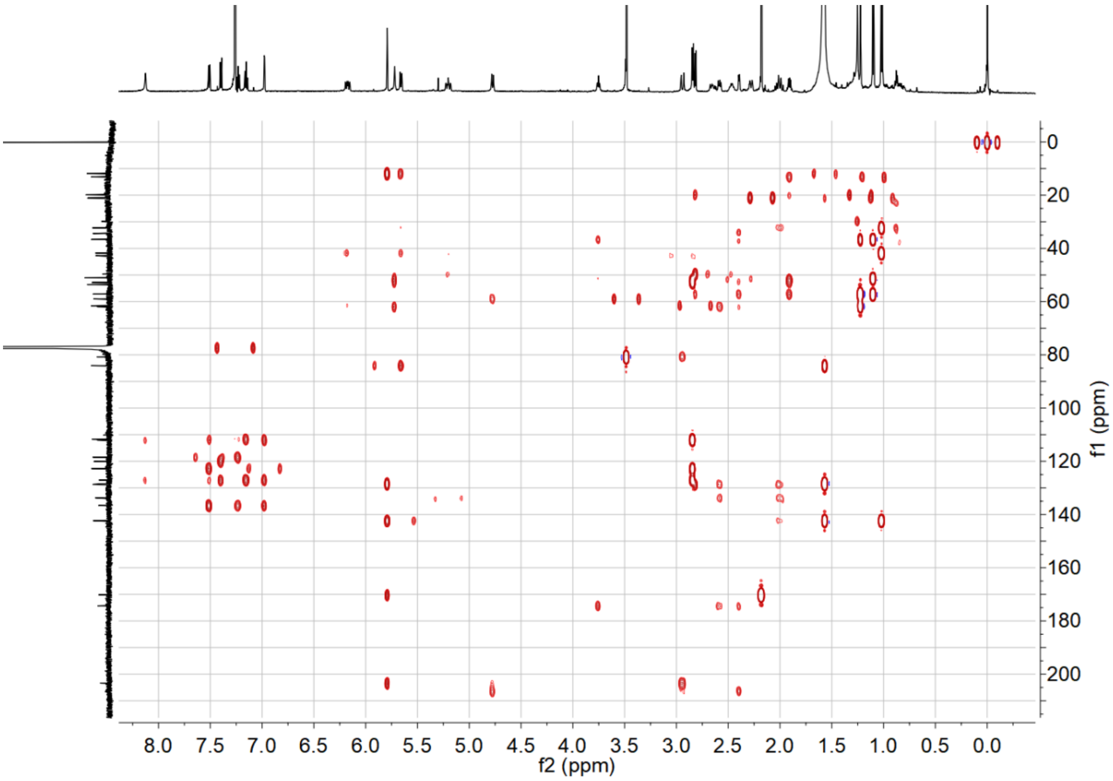

Figure S20. The NOESY spectrum of **3** ($CDCl_3$).

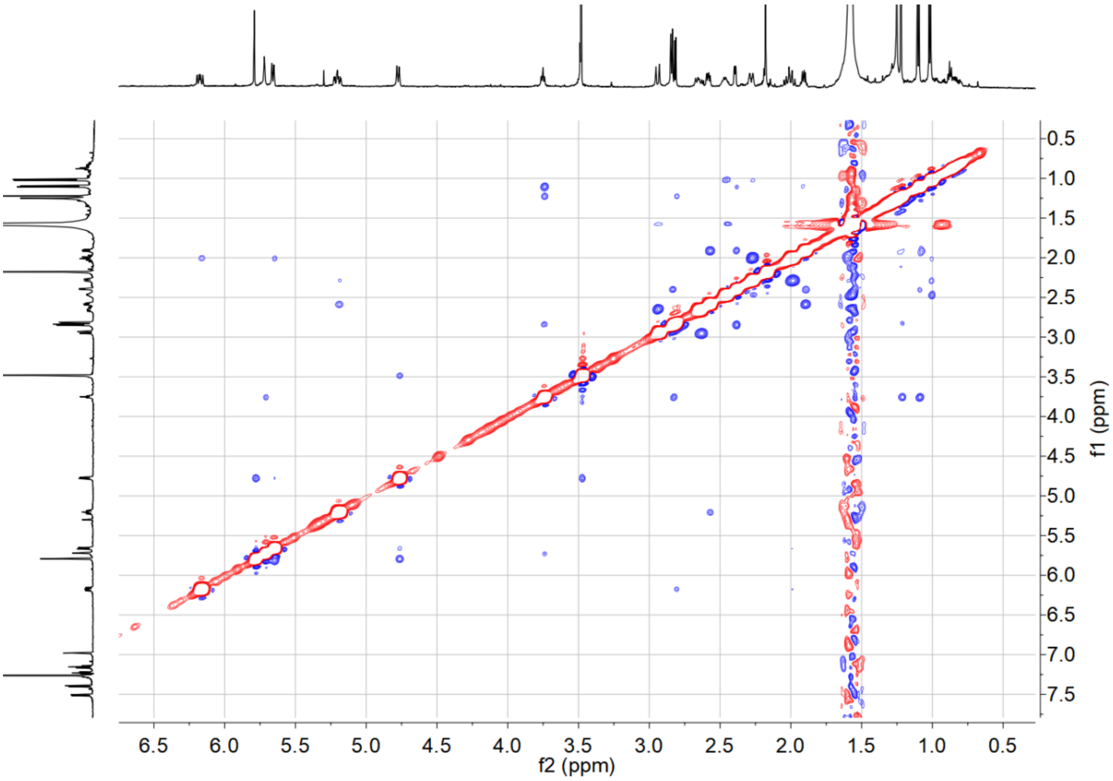

Figure S21. The (+)-HR-ESIMS spectrum of **3**.

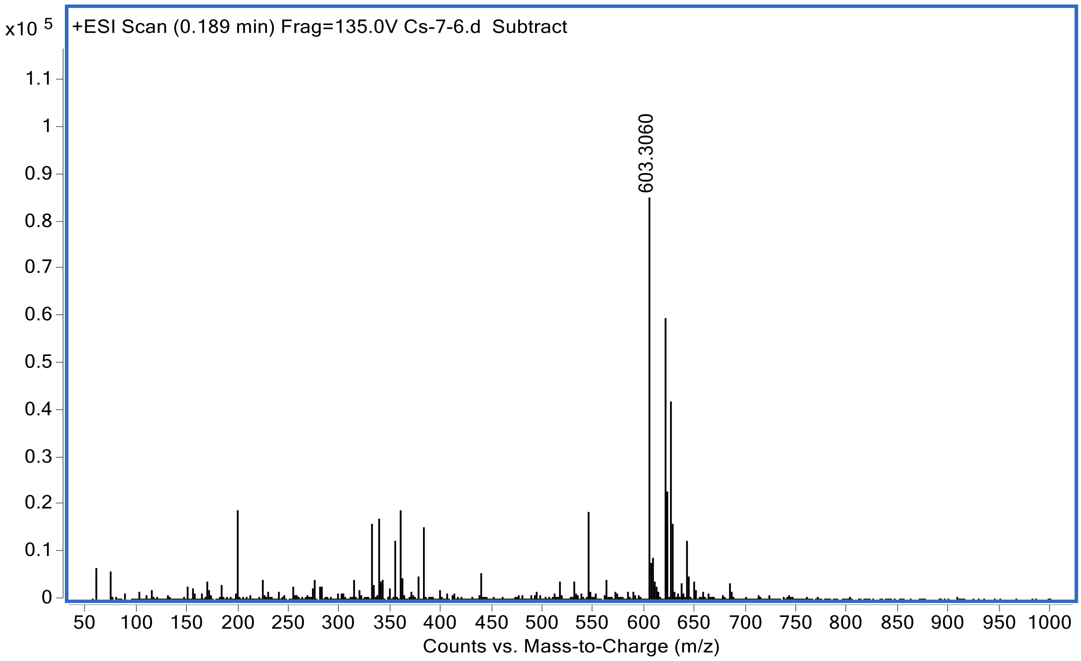

Figure S22. The ^{1}H NMR spectrum of **4** ($CDCl_3$).

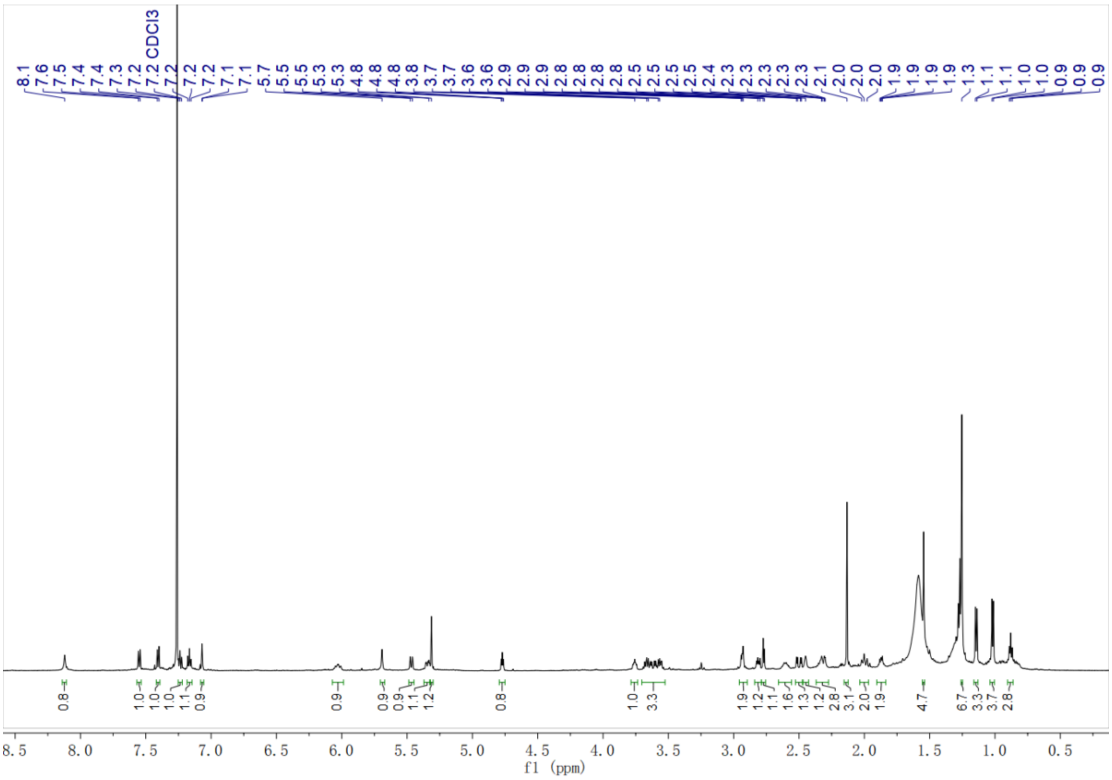

Figure S23. The ^{13}C and DEPT NMR spectra of **4** ($CDCl_3$).

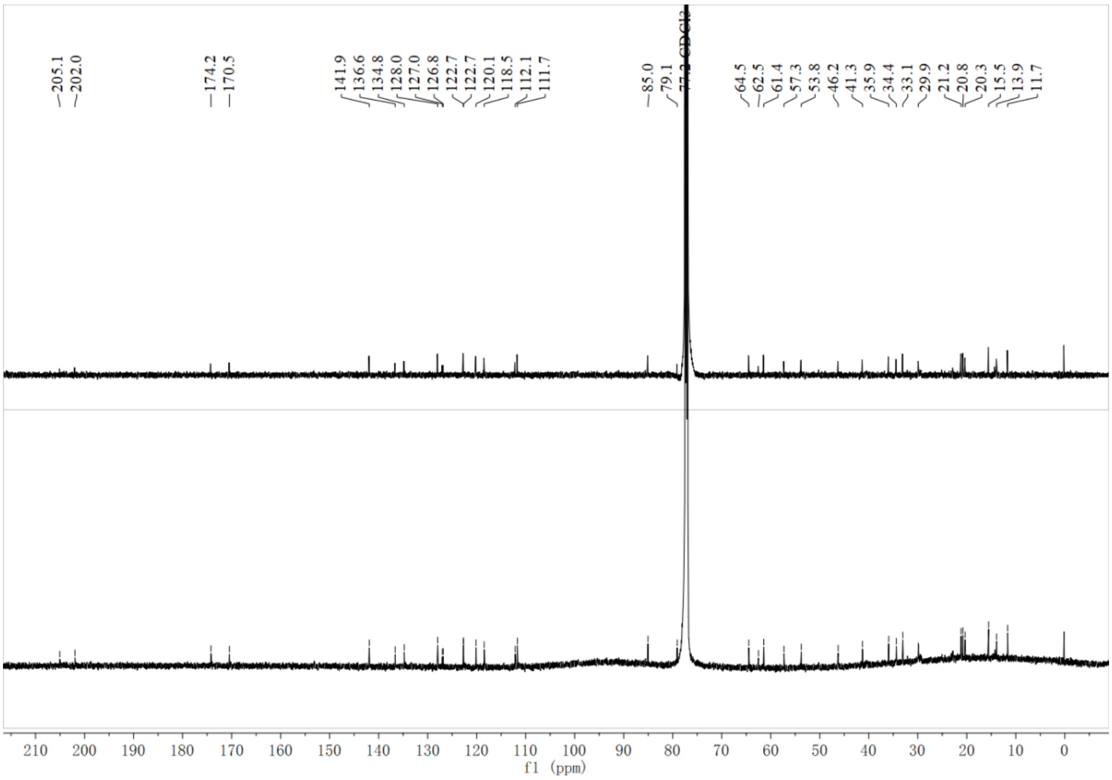

Figure S24. The ^{1}H-^{1}H COSY spectrum of **4** ($CDCl_3$).

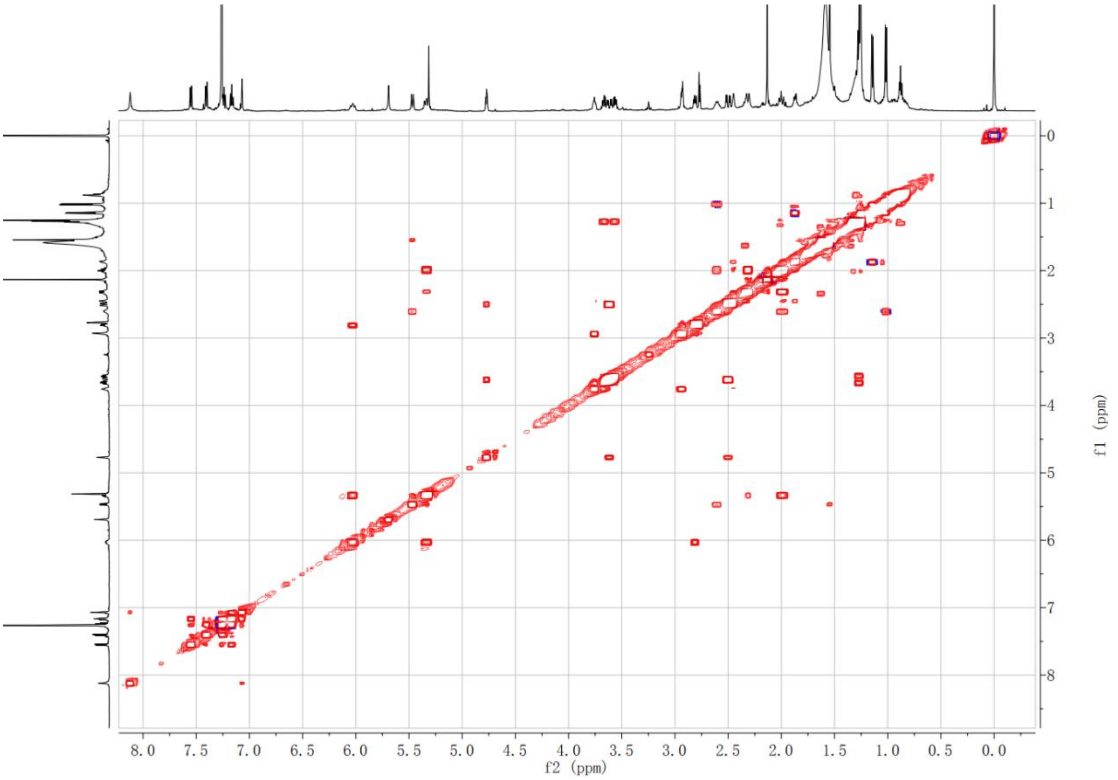

Figure S25. The HSQC spectrum of **4** ($CDCl_3$).

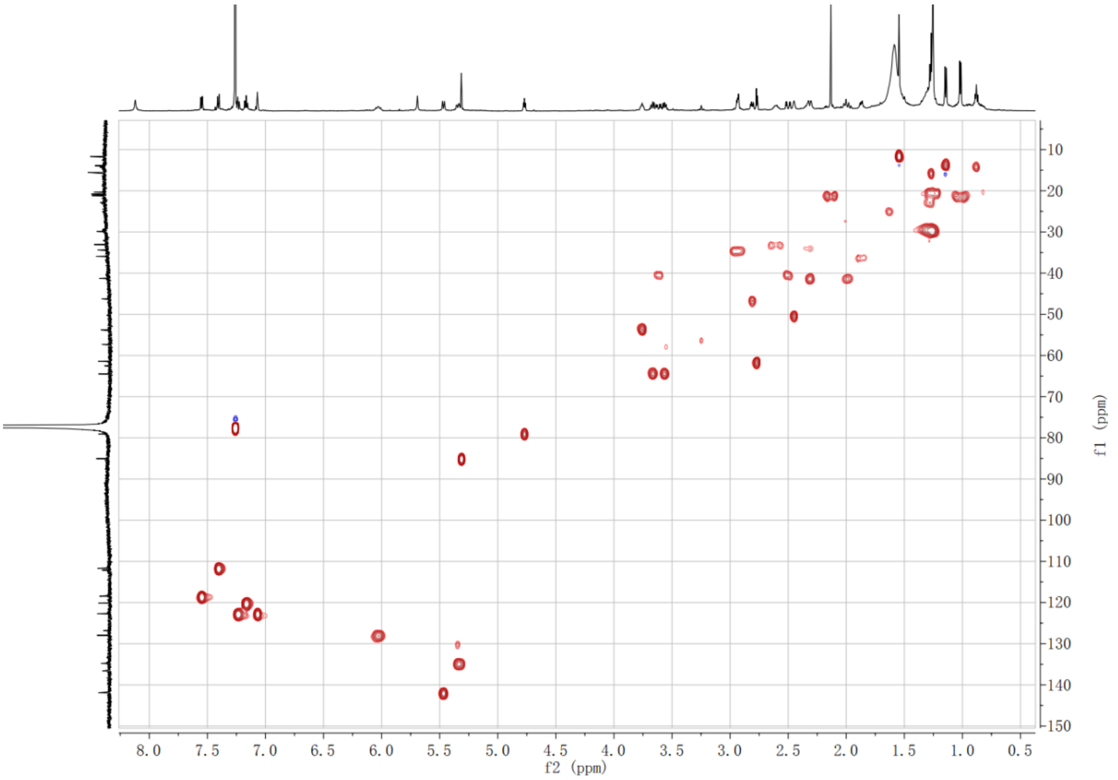

Figure S26. The HMBC spectrum of **4** ($CDCl_3$).

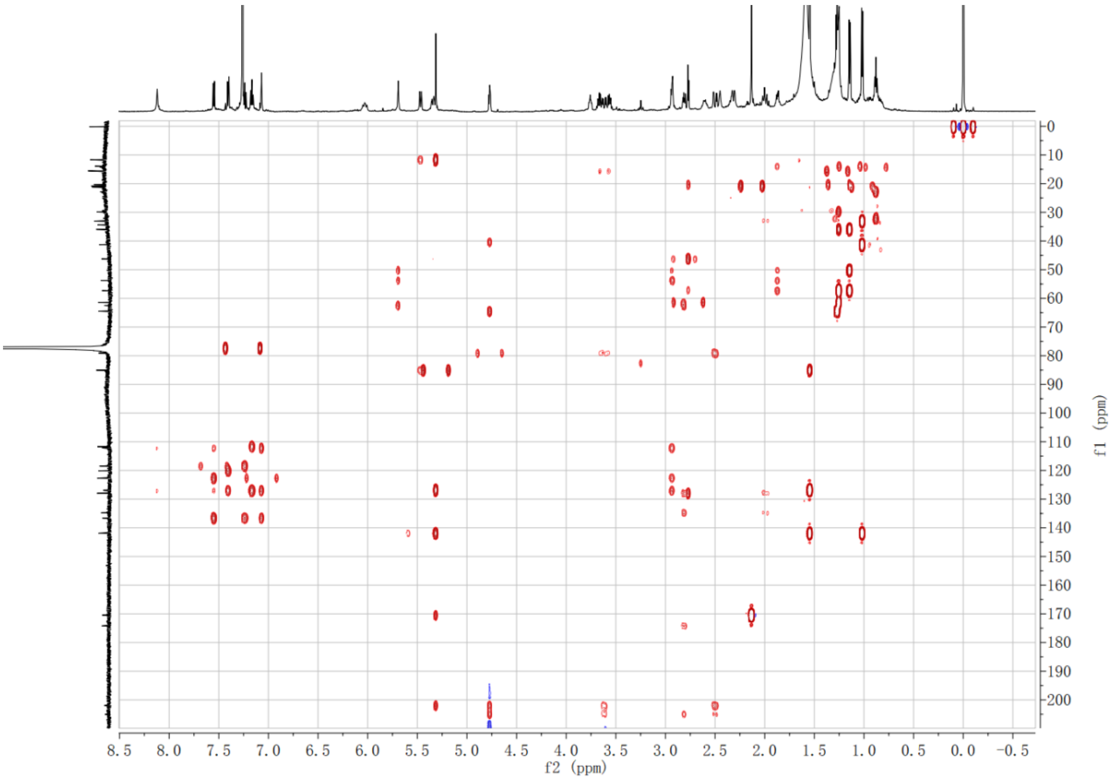

Figure S27. The NOESY spectrum of **4** ($CDCl_3$).

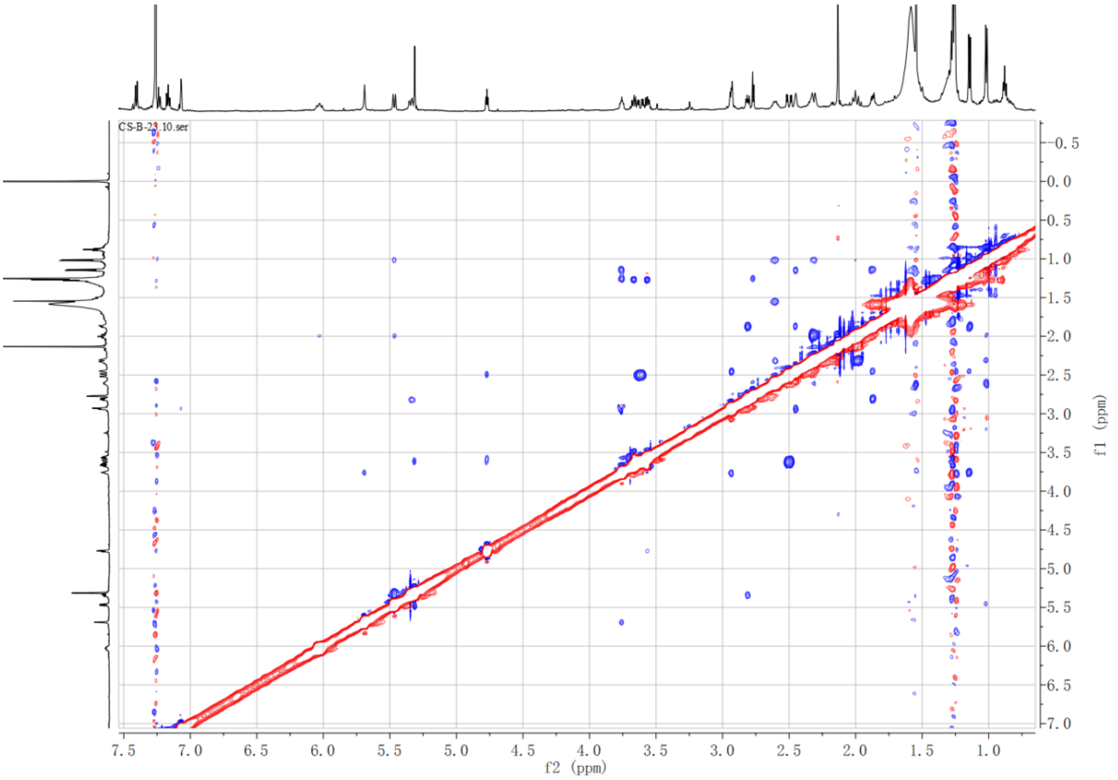

Figure S28. The (+)-HR-ESIMS spectrum of **4**.

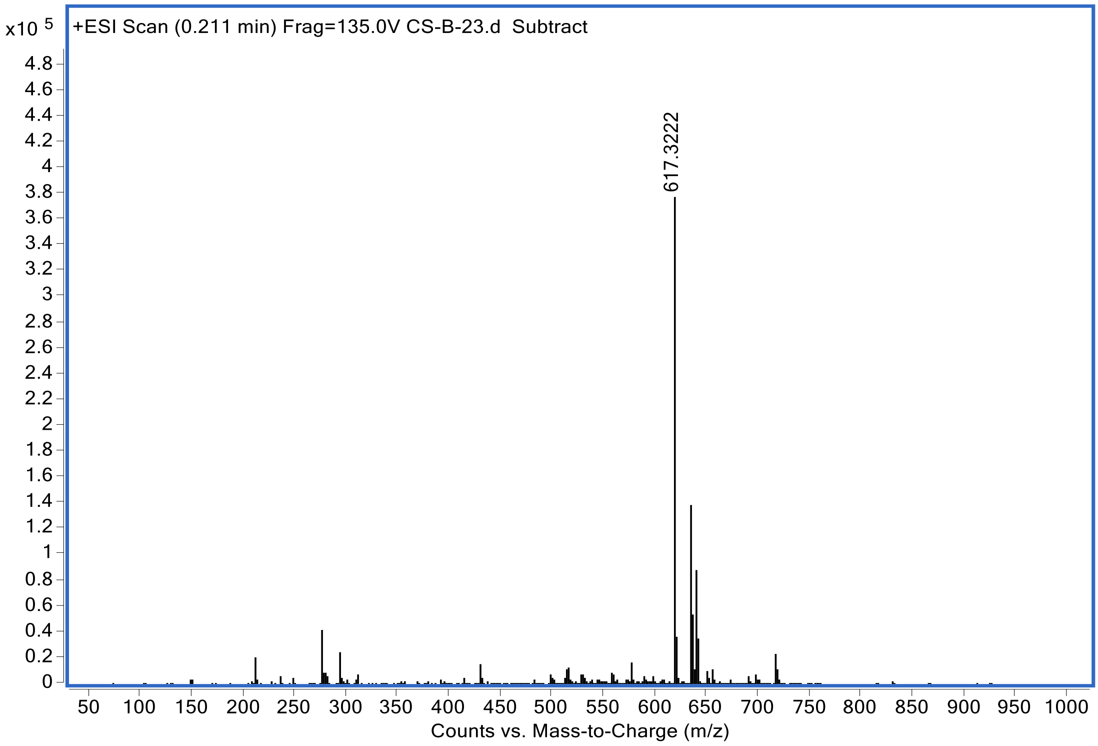

Figure S29. The ^{1}H NMR spectrum of **5** ($CDCl_3$).

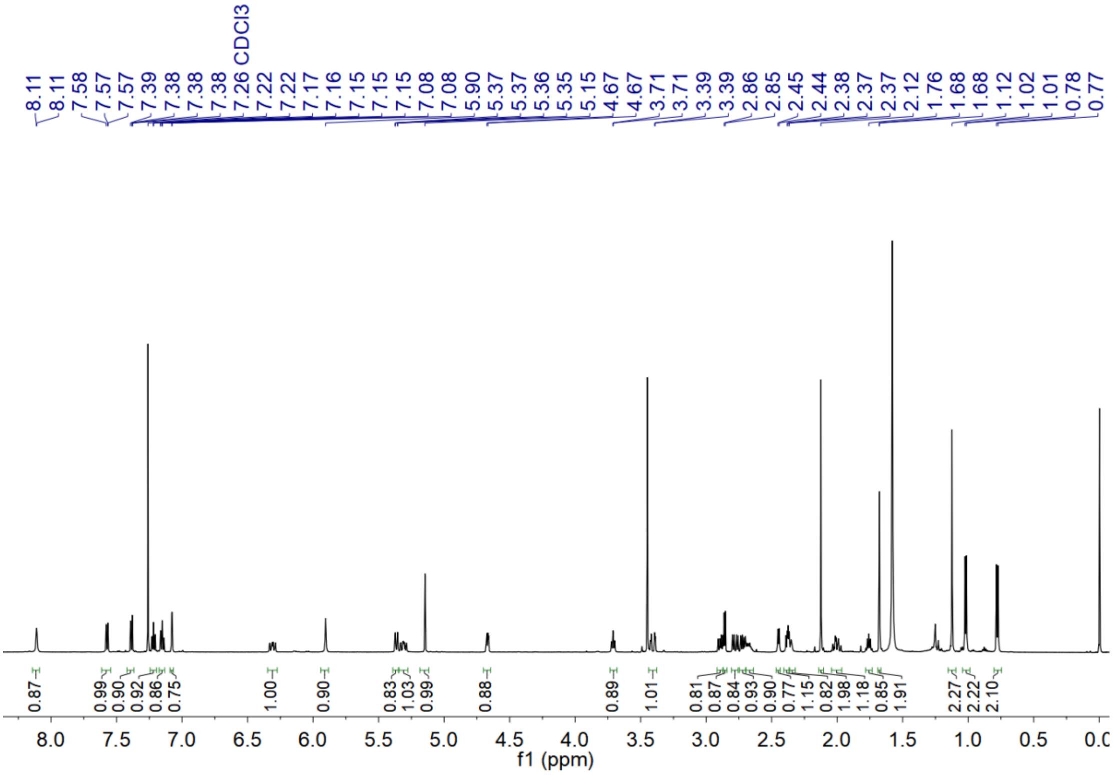

Figure S30. The ^{13}C and DEPT NMR spectra of **5** ($CDCl_3$, 150 MHz).

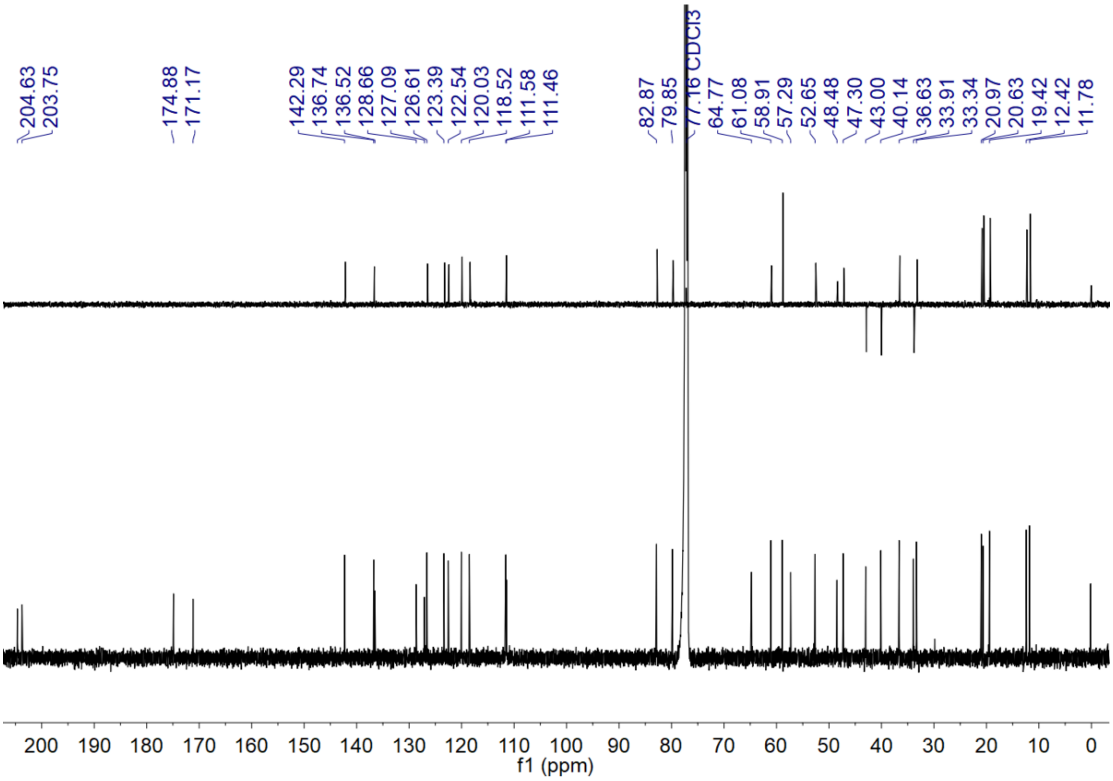

Figure S31. The ^1H-^1H COSY spectrum of **5** ($CDCl_3$).

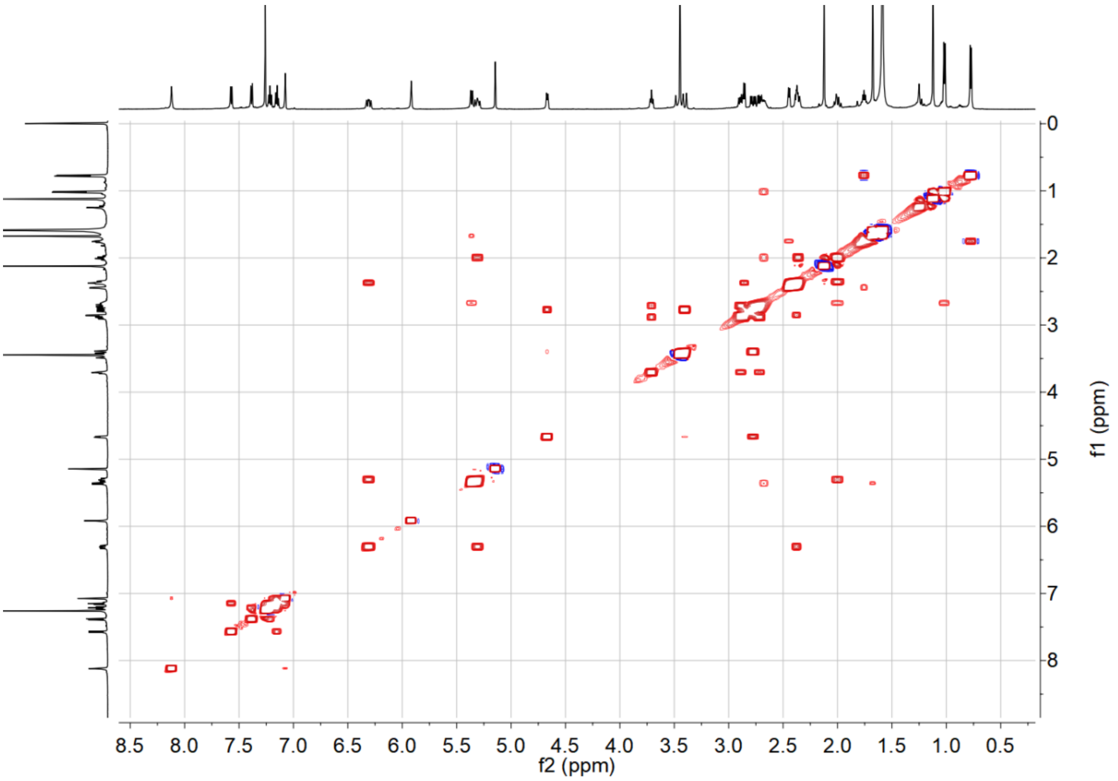

Figure S32. The HSQC spectrum of **5** ($CDCl_3$).

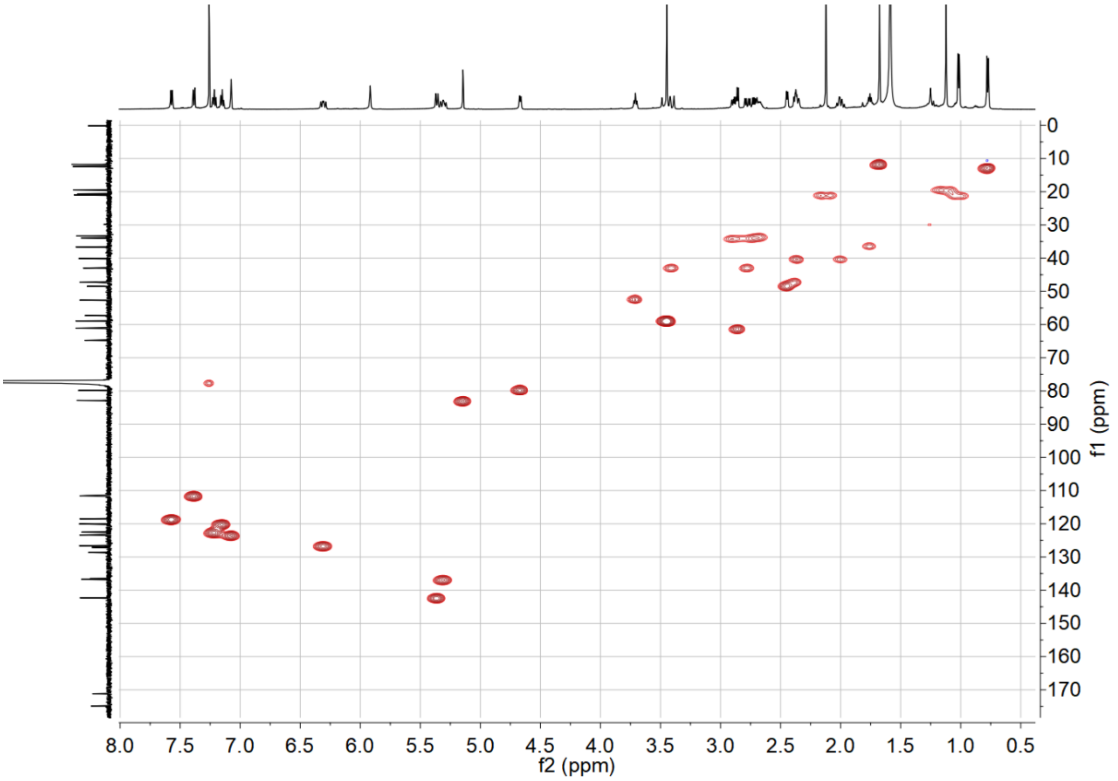

Figure S33. The HMBC spectrum of **5** ($CDCl_3$).

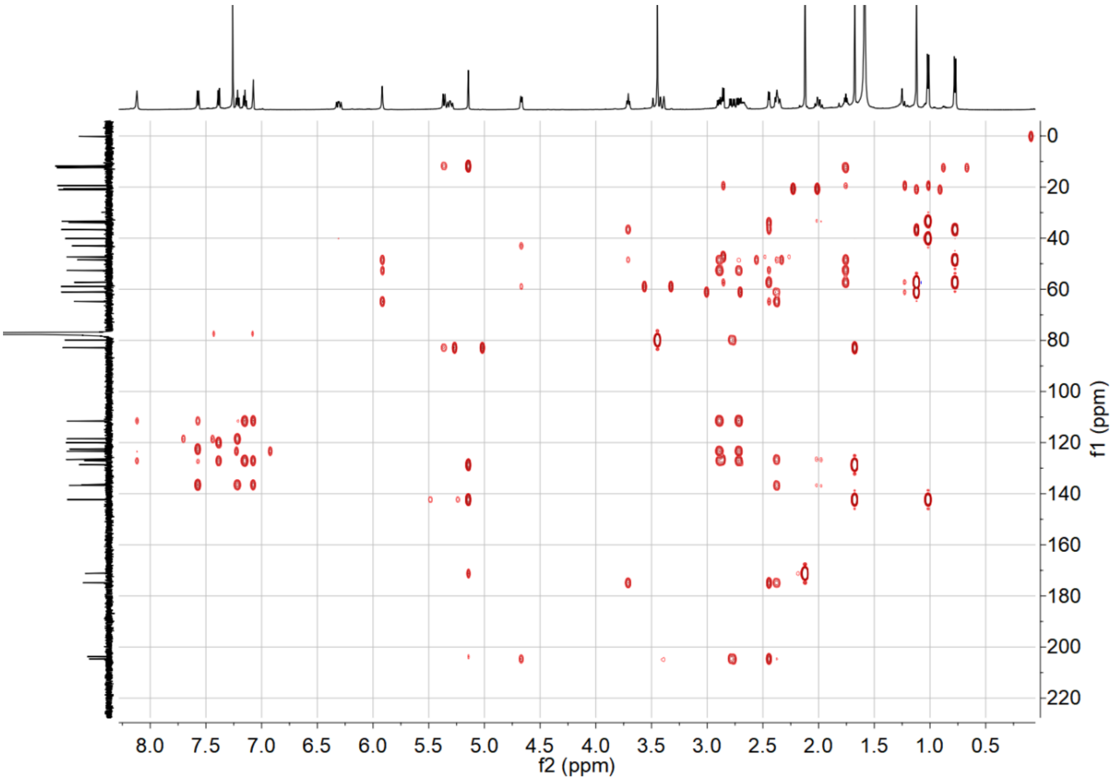

Figure S34. The NOESY spectrum of **5** ($CDCl_3$).

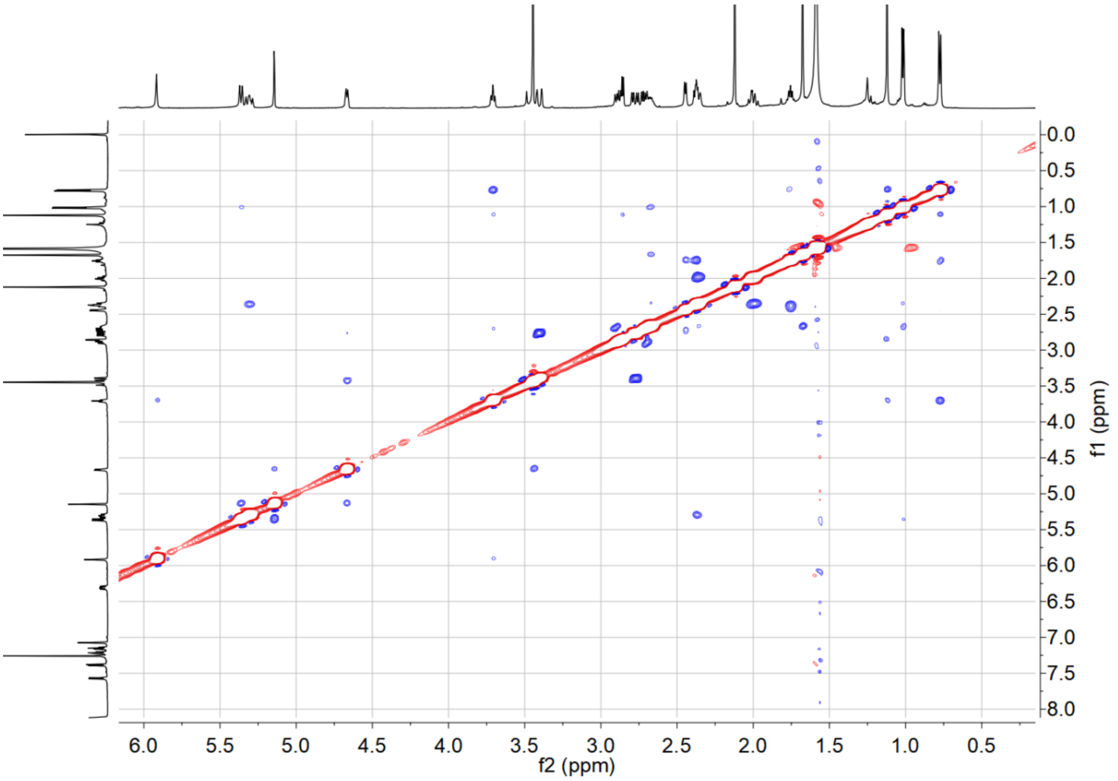

Figure S35. The (+)-HR-ESIMS spectrum of **5**.

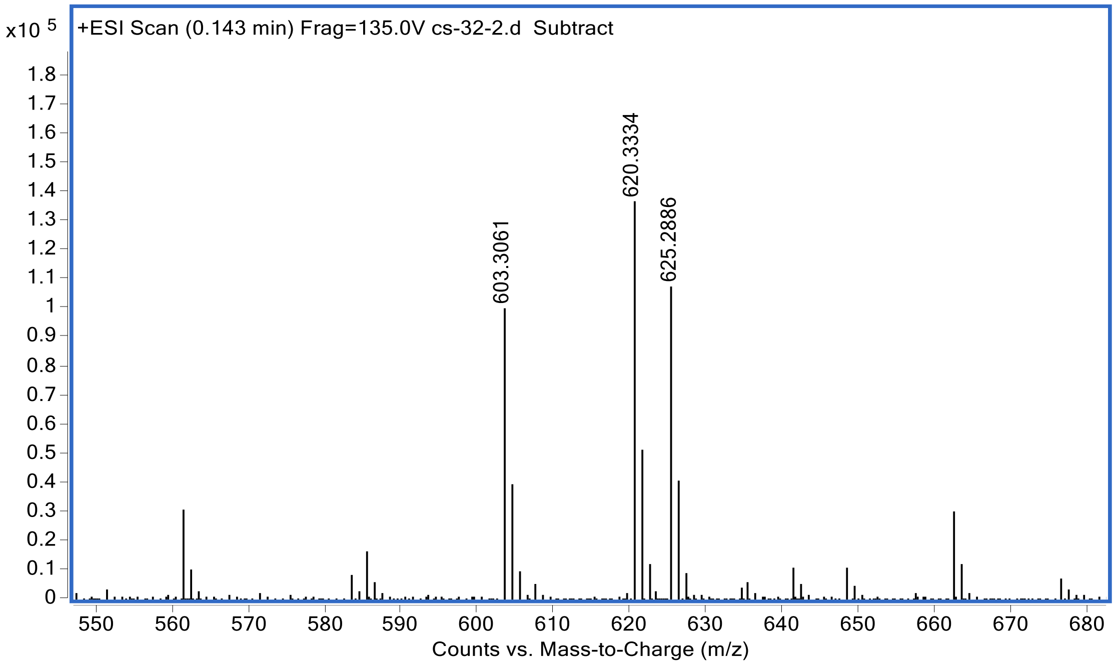

Figure S36. The ^{1}H NMR spectrum of **6** ($CDCl_3$).

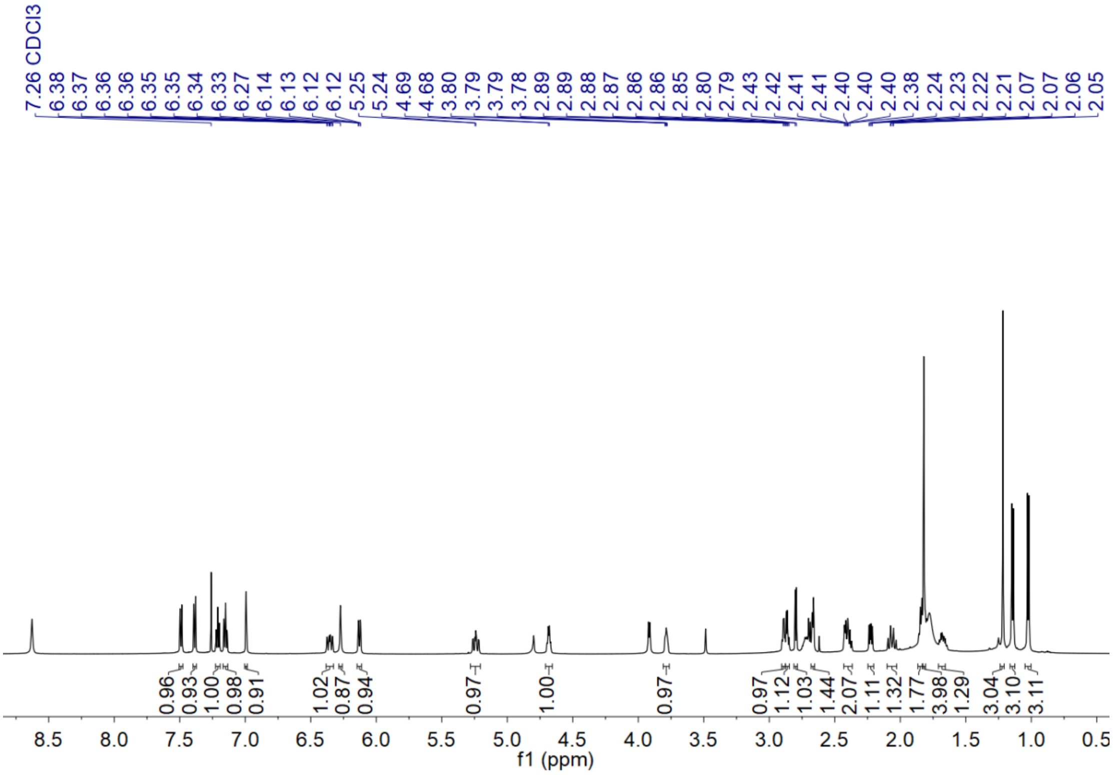

Figure S37. The ^{13}C and DEPT NMR spectra of **6** ($CDCl_3$).

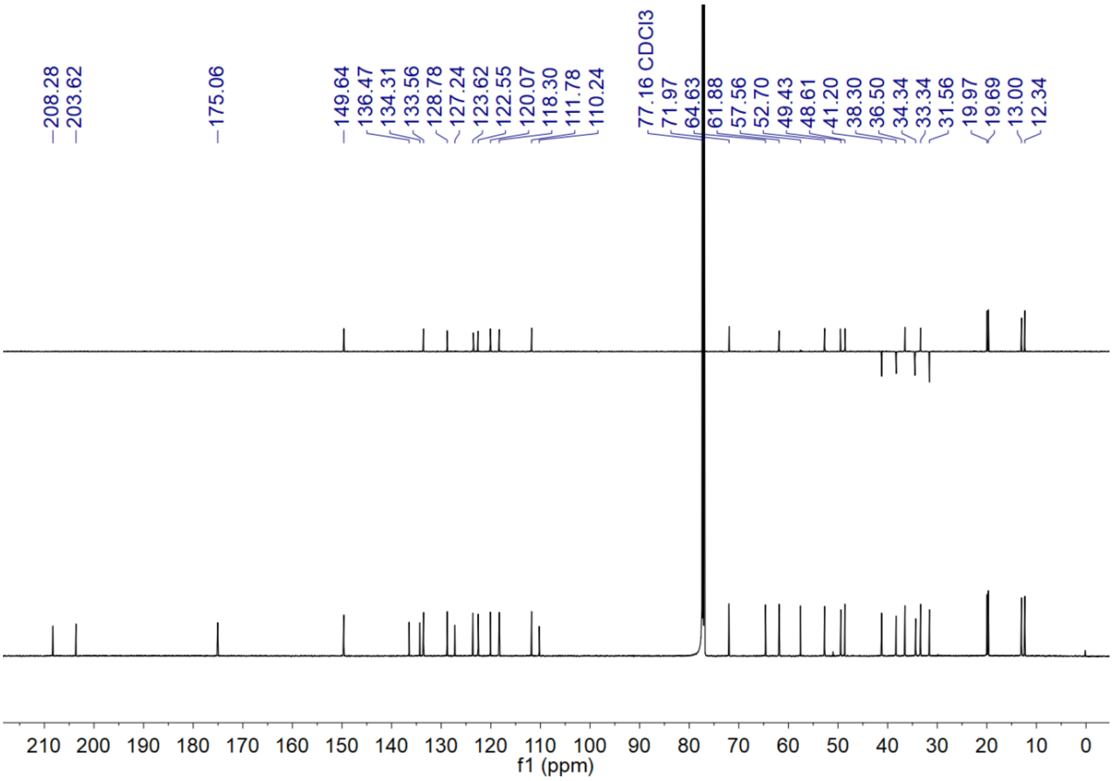

Figure S38. The ^1H NMR spectrum of **7** ($CDCl_3$).

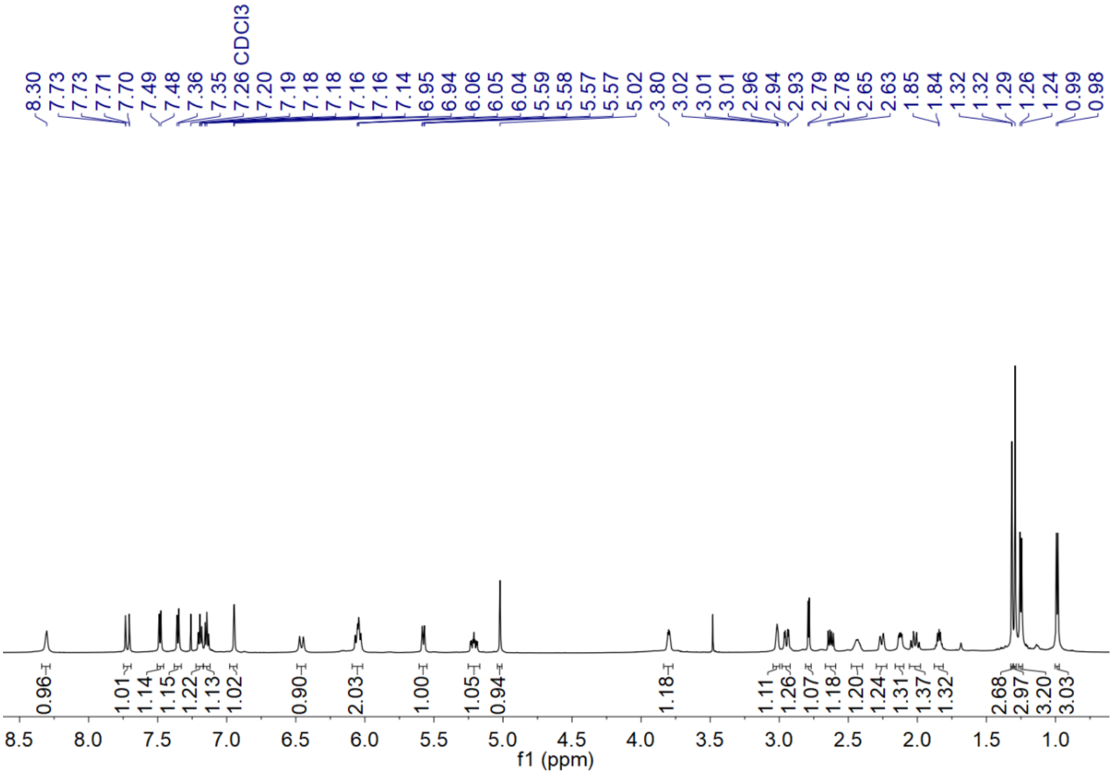

Figure S39. The ^{13}C and DEPT NMR spectra of **7** ($CDCl_3$).

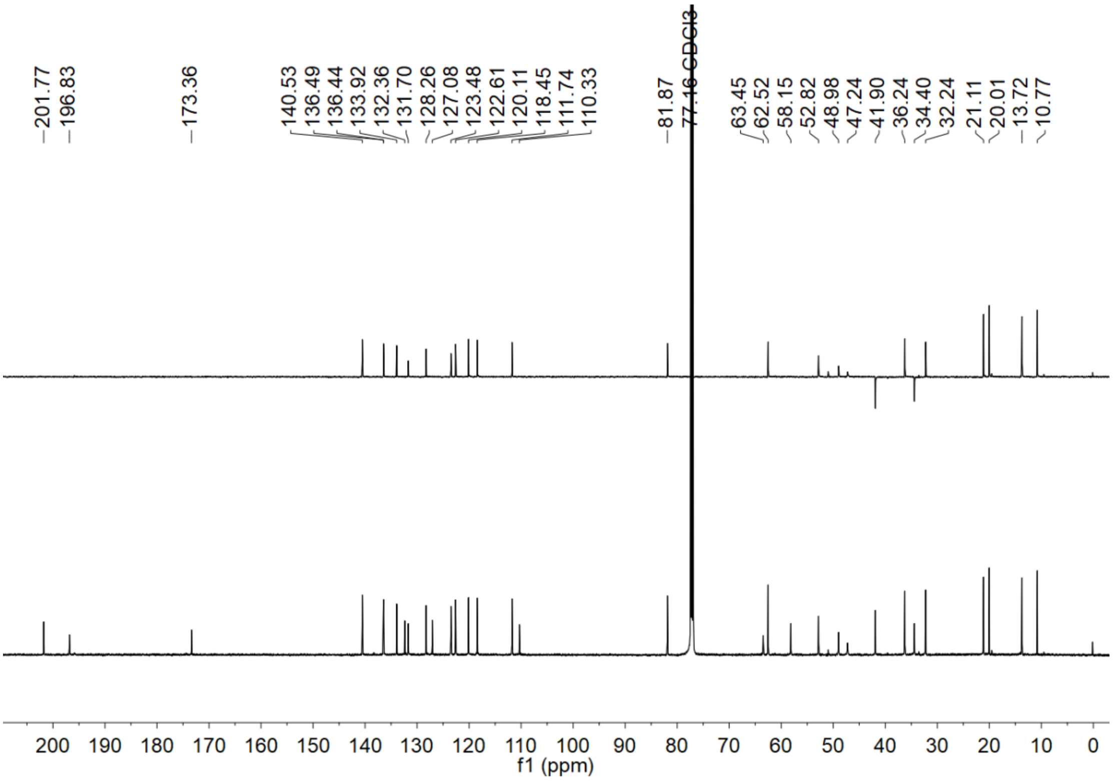

Figure S40. The ^{1}H NMR spectrum of **8** ($CDCl_3$).

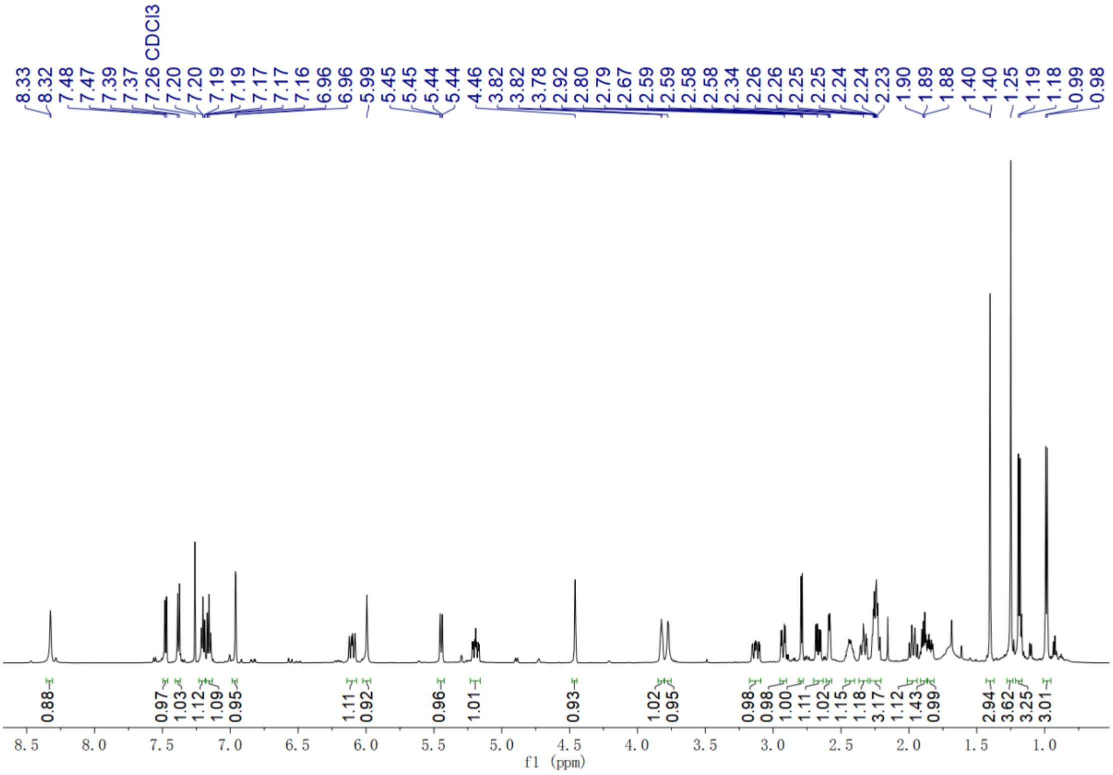

Figure S41. The ^{13}C and DEPT NMR spectra of **8** ($CDCl_3$).

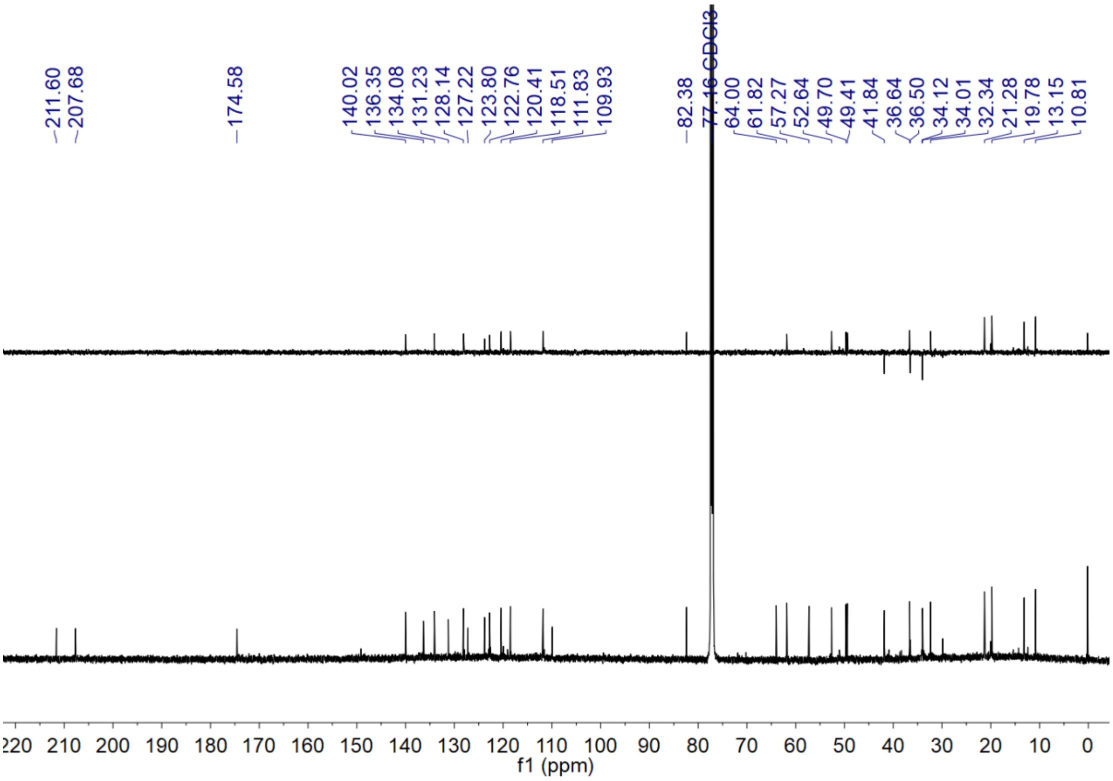

Figure S42. The ^{1}H NMR spectrum of **9** ($CDCl_3$).

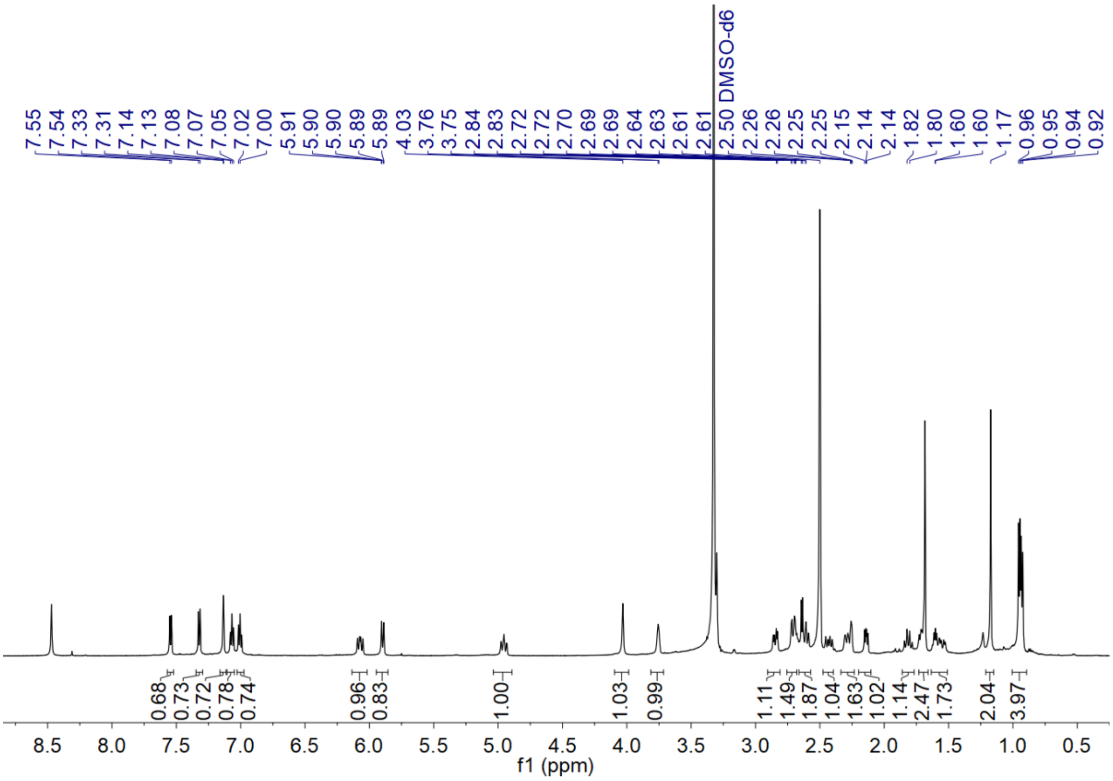

Figure S43. The ^{13}C and DEPT NMR spectra of **9** ($CDCl_3$).

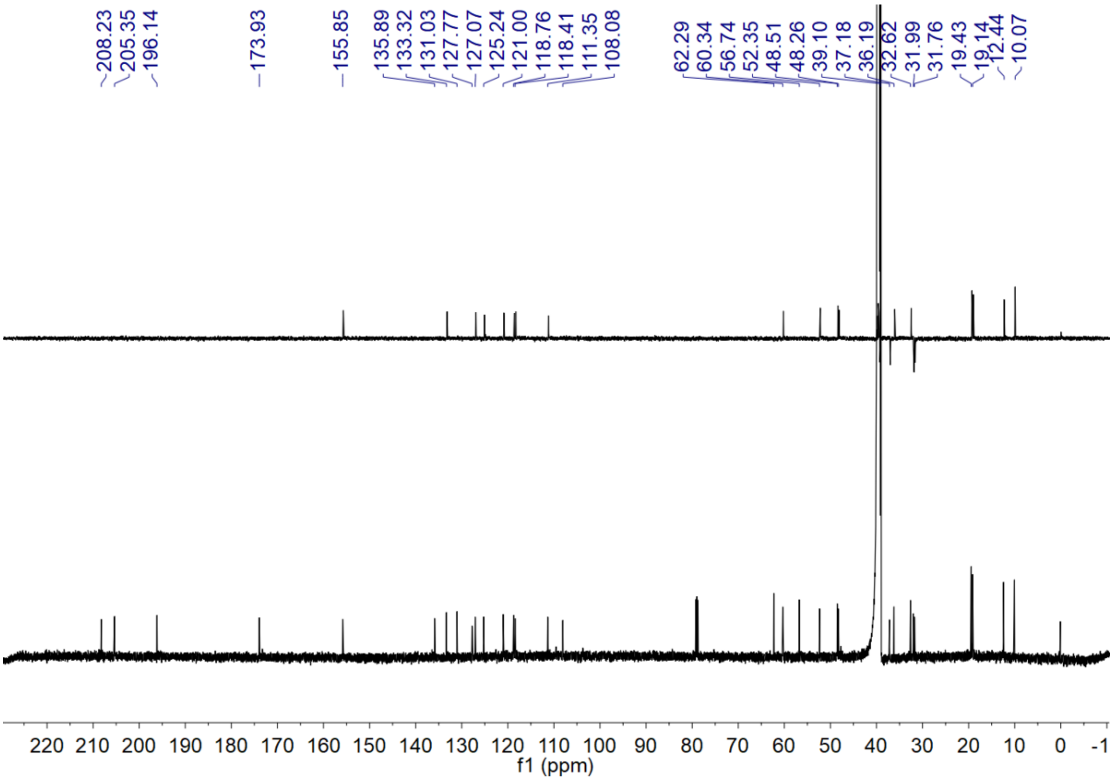

Figure S44. Schematic Diagram of Compound Separation Process

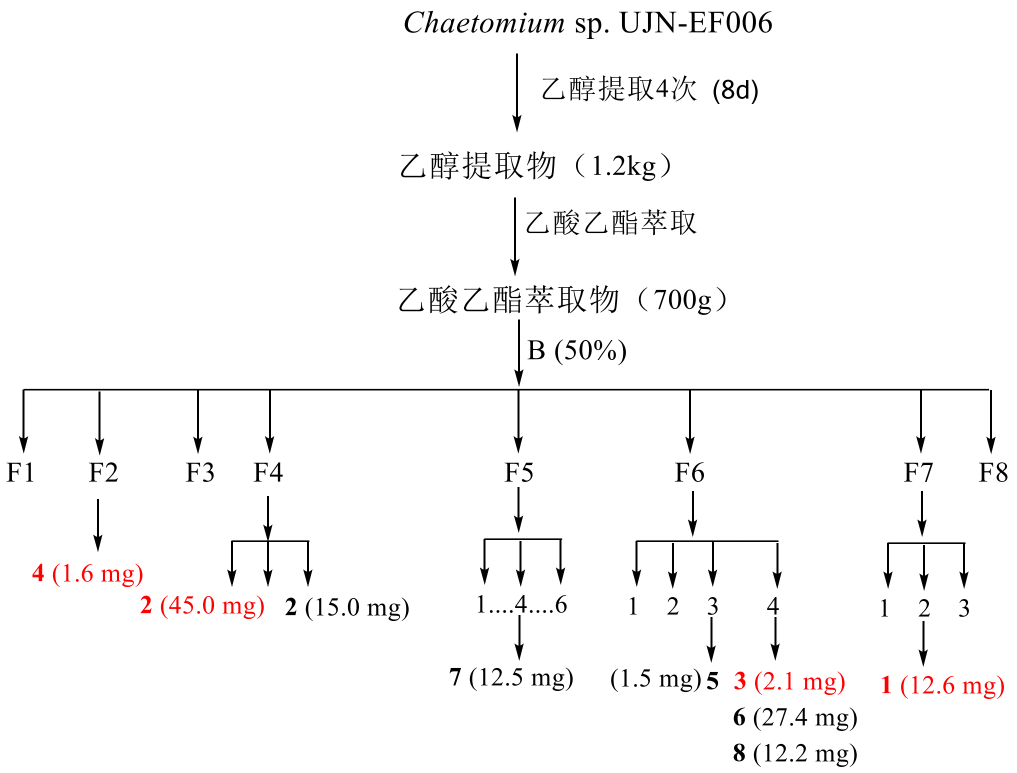

Figure S45. ITS sequencing information of *Chaetomium globosum* and Phylogenetic tree analysis.

>*Chaetomium* sp. UJN-EF006

TTCCTCCGCTTTTGATATGCTTAAGTTCAGCGGGTCTTCCTACCTGATCCG
AGGTCAACCTTGGGTTAAAAGGTGGTTTAACGGCCGGAACCCGCGGCGCG
ACCAGAGCGAGATGTATGCTACTACGCTCGGTGCGACAGCGAGCCCGCCA
CTGCTTTTCAGGGCCTGCGGCAGCCGCAGGTCCCCAACACAAGCCCGGGG
GCTTGATGGTTGAAATGACGCTCGAACAGGCATGCCCGCCAGAATACTGG
CGGGCGCAATGTGCGTTCAAAGATTCGATGATTCACTGAATTCTGCAATTC
ACATTACTTATCGCATTTCGCTGCGTTCTTCATCGATGCCAGAACCAAGAG
ATCCGTTGTTGAAAGTTTTGACTTATTCAGTACAGAAGACTCAGAGAGGC
CATAAATTATCAAGAGTTTGGTGACCTCCGGCGGGCGCCCGCGGTGGGGC
CCAGGGACGCCCGGGGGGTAAACCCCGAGGCCGCCCGCCGAAGCAACGG
TATAGGTAACGTTCACAATGGTTTAGGGAGTTTTGCAACTCTGTAATGATC
CCTCCGCAGGTCAACCTACGGAG

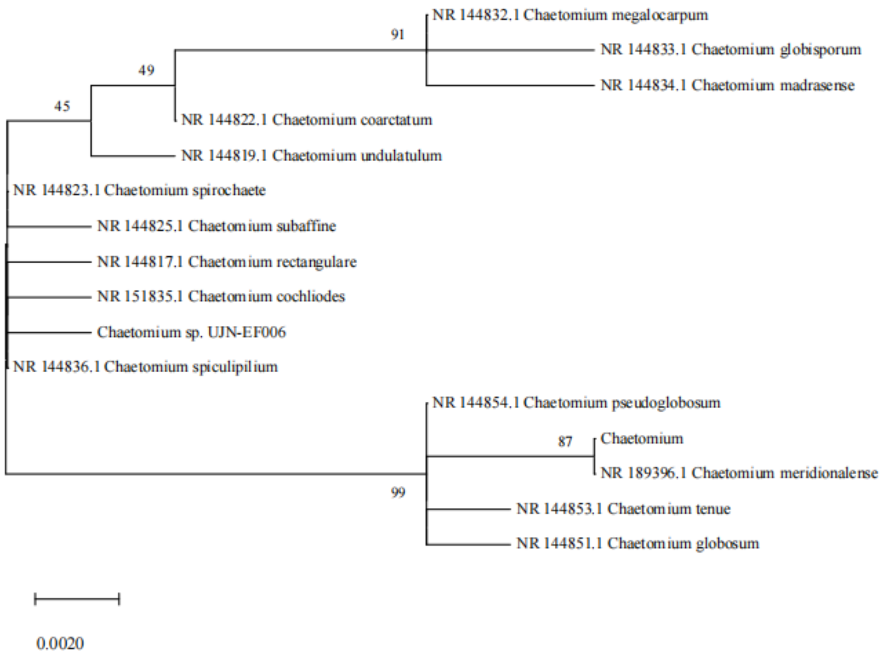

Figure S46. The amplified representative pictures of the hypha morphology of *B. cinerea* observed under SEM.

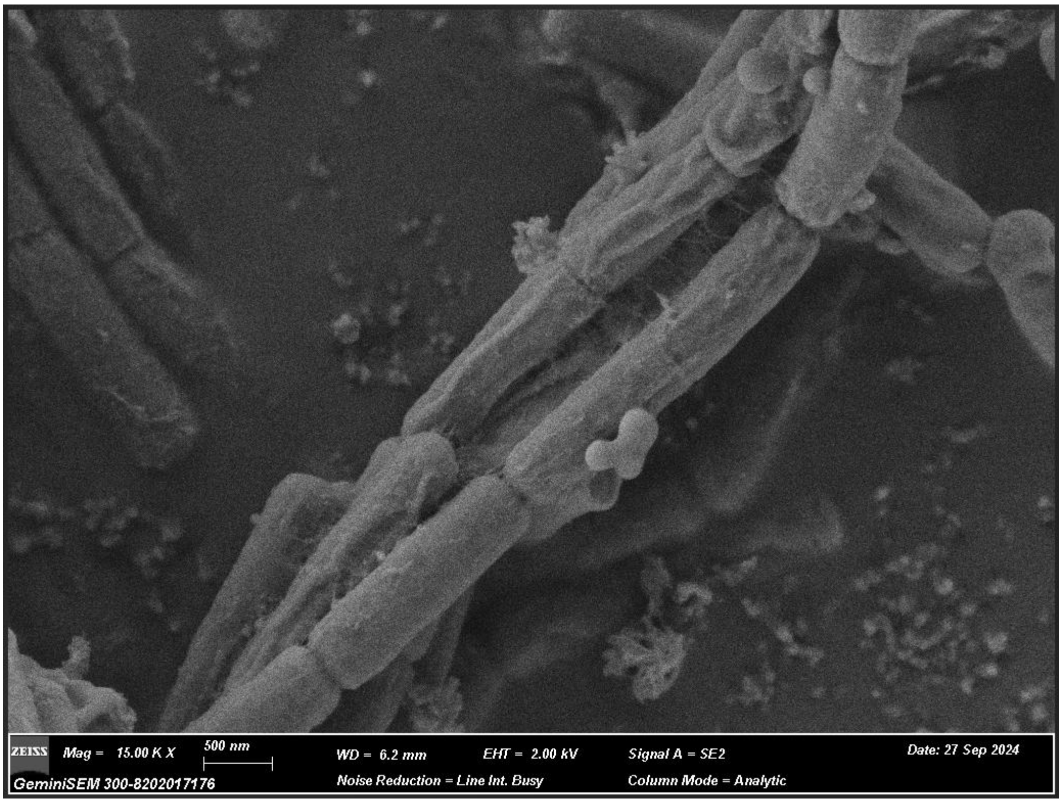

(**A**) Blank control with 1% DMSO (15.00 k×, 0.5 μm);

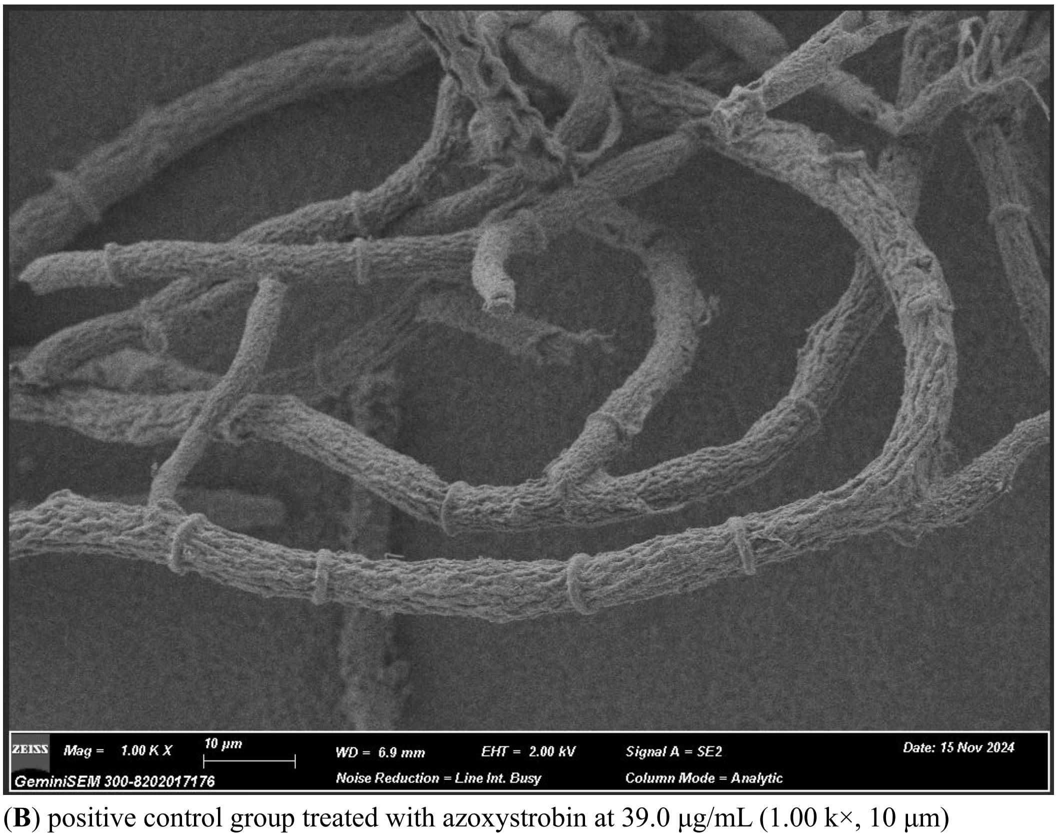

(**B**) positive control group treated with azoxystrobin at 39.0 μg/mL (1.00 k×, 10 μm)

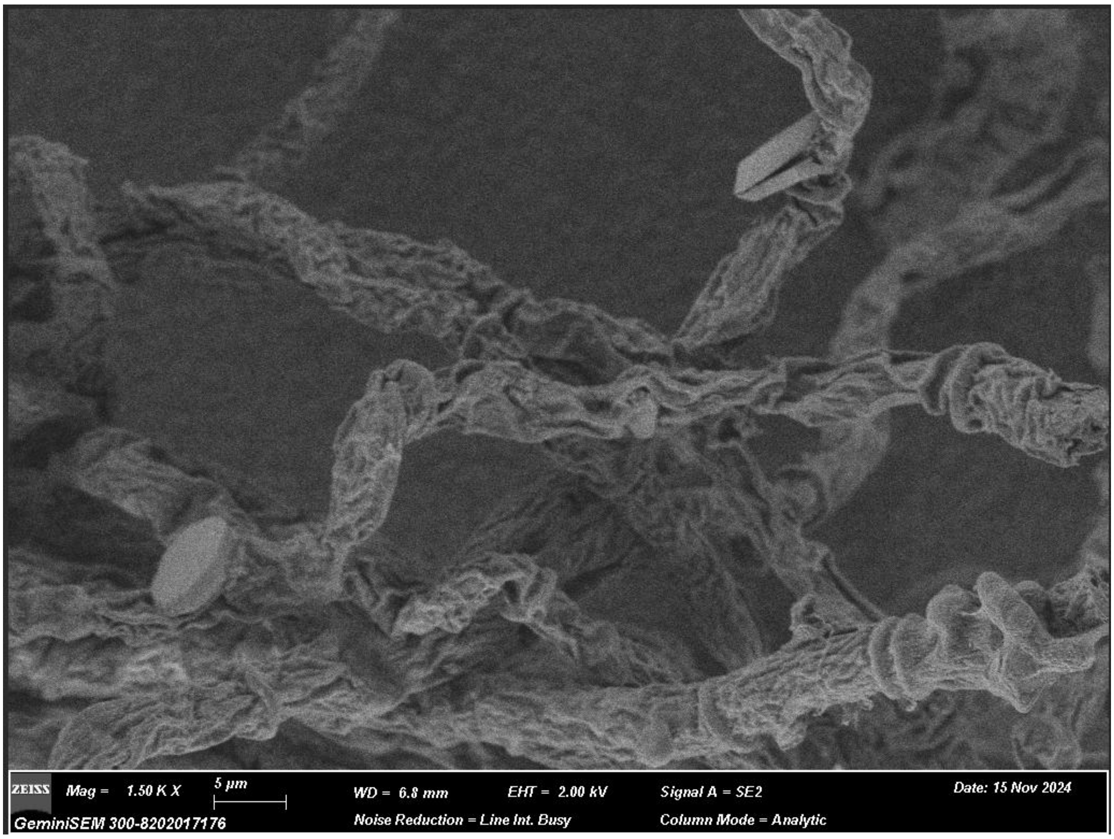

(**C**) treated group with compound **2** at 2.5 µg/mL (1.50 k×, 5 µm)

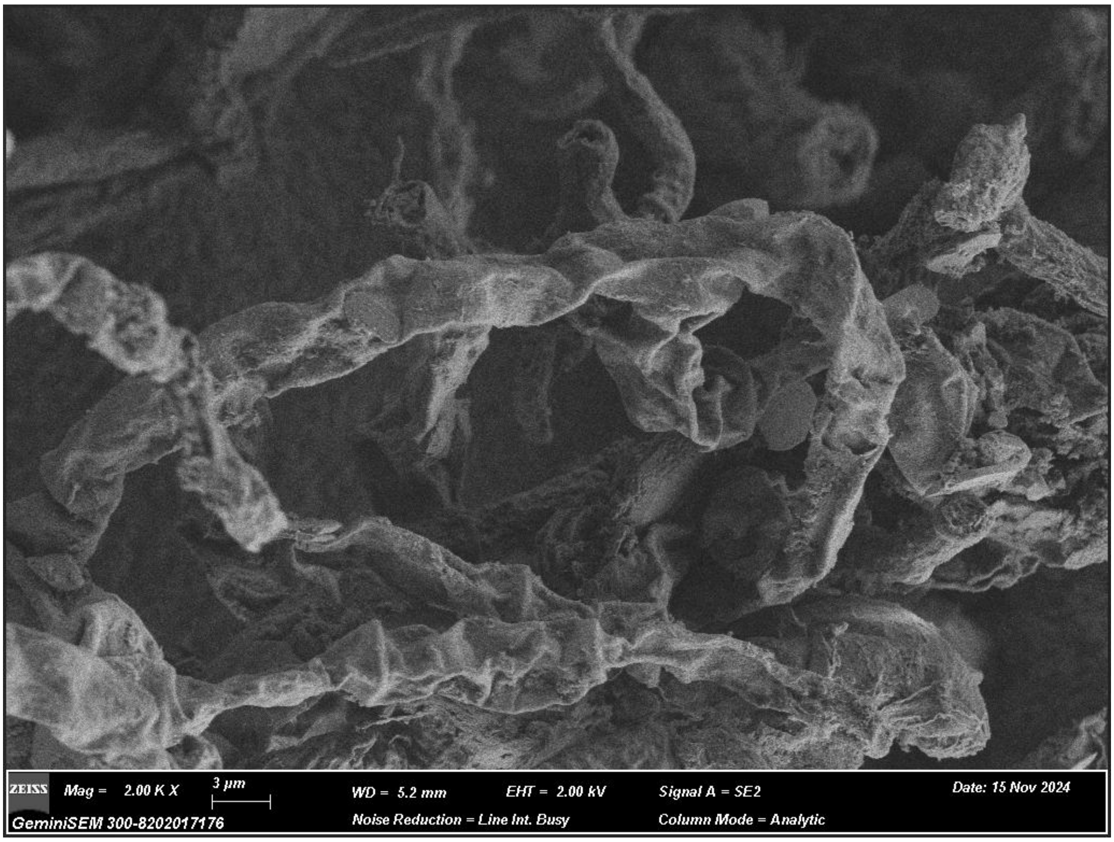

(**D**) treated with **2** at 5.0 µg/mL (2.00 k×, 3 µm)
